# Serendipity discrete complexes with enhanced regularity

**DOI:** 10.1007/s10092-025-00665-w

**Published:** 2025-10-15

**Authors:** Daniele A. Di Pietro, Marien Hanot, Marwa Salah

**Affiliations:** 1 IMAG, Univ Montpellier, CNRS, Montpellier, France; 2 LPP, University of Lille, CNRS, Inria, Lille, France

**Keywords:** Discrete de Rham method, Compatible discretizations, Serendipity, Rot–rot complex, Stokes complex, 65N30, 65N99, 65N12, 35Q60

## Abstract

In this work we address the problem of finding serendipity versions of approximate de Rham complexes with enhanced regularity. The starting point is a new abstract construction of general scope which, given three complexes linked by extension and reduction maps, generates a fourth complex with cohomology isomorphic to the former three. This construction is used to devise new serendipity versions of rot–rot and Stokes complexes derived in the Discrete de Rham spirit.

## Introduction

The well-posedness of important classes of partial differential equations hinges on the algebraic and analytical properties of the de Rham complex [1] which, for a domain $\Omega \subset \mathbb{R}^3$, reads1.1Compatible discretizations are built mimicking these properties at the discrete level in order to achieve stability; see, e.g., the seminal works [7, 14, 17, 20, 21, 29–31]. Arbitrary-order counterparts of (1.1) on general polytopal meshes have been recently derived in [22, 23, 25] following the virtual element approach and in [11, 13] following the fully discrete approach; see also [6] for a recent extension to the de Rham complex of differential forms.

The standard de Rham complex (1.1), however, does not encode sufficient regularity for certain applications, and variants with enhanced regularity have to be considered. A first example is provided by fourth-order problems appearing in electromagnetism, for which the relevant complex is the *rot-rot complex*:1.2where $\Omega$ is a domain of $\mathbb{R}^2$, $\boldsymbol{H}({\textbf{rot}}{{\,\mathrm{rot}\,}};\Omega )$ is the $L^2$-graph space of the ${\textbf{rot}}{{\,\mathrm{rot}\,}}$ operator where, for smooth enough functions $q: \Omega \rightarrow \mathbb{R}$ and $$\boldsymbol{v} = \begin{pmatrix} v_1 \\ v_2\end{pmatrix}: \Omega \rightarrow \mathbb{R}^2$$, $${\textbf{rot}}q {:}{=}\begin{pmatrix} \partial _2 q \\ -\partial _1 q\end{pmatrix}$$ and ${{\,\mathrm{rot}\,}}\boldsymbol{v} {:}{=}\partial _1 v_2 - \partial _2 v_1$. Another example, relevant to incompressible flow problems, is the *Stokes complex* which, for a domain $\Omega \subset \mathbb{R}^3$, reads1.3Polytopal approximations of the above and related complexes have been developed in [8, 10, 19]. We also refer to [3] for a discussion on their links with the standard de Rham complex.

Recent works [12, 24, 26, 28] have pointed out the possibility to apply *serendipity* techniques to discrete de Rham complexes on polytopal meshes. Such techniques reduce the number of internal degrees of freedom (DOFs) by reconstructing the missing bits of information from boudary DOFs while preserving the degree of polynomial consistency; see also [9, 15, 16] for similar ideas in the context of lowest-order methods. Devising serendipity versions of discrete complexes with enhanced regularity discretizing, e.g., (1.2) or (1.3) is, to date, an open problem. Such serendipity versions could crucially reduce the computational cost in certain situations. For the three-dimensional Stokes complex (1.3), e.g., they lead to the elimination of (certain) internal face DOFs, which is not possible by static condensation. In the context of non-linear problems in any space dimension, they are a much more effective means to reduce the size of the corresponding algebraic problem when compared to static condensation, as they do not require additional computation at each non-linear iteration.

The aim of this work is precisely to fill this gap. Specifically, two crucial advances with respect to the previous literature can be identified:

- We devise a construction of general scope that, given a discrete complex with enhanced regularity and a serendipity version of the underlying de Rham complex, generates a serendipity version of the former with the correct cohomology and the same degree of polynomial consistency;
- We apply the construction to derive the first serendipity version of the Stokes complex of [19] and provide a rigorous justification (and numerical validation) of the serendipity rot-rot complex hinted to in [8, Section 3.6]. The interest of these serendipity complexes is that they offer accuracy comparable to their non-serendipity version for a fraction of the cost.

It is worth emphasizing a crucial difference between the polytopal constructions considered here and classical finite elements. A key ingredient in finite elements are function spaces (typically spanned by polynomials) for which the DOFs are unisolvent. For classical (quadrilateral) serendipity elements, the approximation properties of such spaces can be significantly reduced or lost altogether on distorted meshes; see the insightful works [2, 5] for a discussion on this subject. In polytopal methods, on the other hand, function spaces are replaced by operator reconstructions (or projections in the virtual element wording). Such reconstructions are built working directly on the DOFs and encode polynomial consistency even on distorted meshes, i.e., they are exact when applied to the interpolates of polynomial functions up to a certain degree. The framework proposed here ensures that the polynomial consistency properties of the underlying de Rham complex are inherited by the corresponding discrete complex with enhanced regularity.

A second aspect worth mentioning is that applying serendipity techniques to a discrete complex is significantly more difficult than working on a single space, as one must make sure that the elimination of DOFs does not alter its homological properties; see, e.g., [4, 18] for recent developments in the context finite elements. Compatible serendipity techniques to reduce the number of face DOFs in virtual element discretizations of the de Rham complex have been developed in [23, 28], where a direct proof of local exactness properties was provided. A variation of the discrete complex in the previous reference has been recently proposed in [27], where links with Discrete de Rham (DDR) methods have also been established. A systematic approach to serendipity for polyhedral approximations of discrete complexes, including the elimination of both element and face DOFs, has been recently proposed in [12] and applied to the DDR complex of [11].

The rest of this work is organized as follows. In Sect. 2 we present the abstract construction. The discrete de Rham complex of [11] along with its serendipity version of [12] are briefly recalled in Sect. 3. Serendipity versions of the rot-rot complex of [8] and of the Stokes complex of [19] are derived and studied in Sects. 4 and 5, respectively. Section 4 also contains numerical experiments comparing the performance of the serendipity and original rot-rot complexes on a quad-rot problem.

## An abstract framework for serendipity complexes with enhanced regularity

### Setting

We consider the situation depicted in the following diagram, involving three complexes $(W_i,\partial _i)_i$, $(\widehat{W}_i,\widehat{\partial _i})_i$, and $(V_i,d_i)_i$ linked by linear extension and reduction operators:2.1In the applications considered considered in Sects. 5 and 4: $(W_i,\partial _i)_i$ is a discretization of the de Rham complex (1.1) or of its two-dimensional counterpart; $(\widehat{W}_i,\widehat{\partial _i})_i$ is a serendipity version of the above; $(V_i,d_i)_i$ is a discretization of a complex with enhanced regularity such as (1.2) or (1.3), typically obtained by an enrichment of $(W_i,\partial _i)_i$. Our goal is to construct a fourth complex that is a serendipity version of $(V_i,d_i)_i$ with the same polynomial consistency and cohomology.

#### Assumption 1

(*Properties of* $E_{{W}_{i}}$ and $\widehat{R}_{{W}_{i}}$) The extension $E_{{W}_{i}}\,:\,\widehat{W}_i \rightarrow W_i$ and reduction $\widehat{R}_{{W}_{i}}\,:\,W_i \rightarrow \widehat{W}_i$ are such that

1. $$(\widehat{R}_{{W}_{i}}E_{{W}_{i}})_{|{{\,\mathrm{Ker}\,}}\widehat{\partial }_i} =\mathrm{Id}_{{{\,\mathrm{Ker}\,}}\widehat{\partial }_i} $$.
2. $$(E_{{W}_{i+1}}\widehat{R}_{{W}_{i+1}}-\mathrm{Id}_{W{i+1}})({{\,\mathrm{Ker}\,}}\partial _{i+1}) \subset \mathrm{Im}(\partial _i)$$.
3. $$\widehat{R}_{{W}_{i+1}} \partial _i=\widehat{\partial }_i\widehat{R}_{{W}_{i}}$$ and $$E_{{W}_{i+1}}\widehat{\partial }_i= \partial _i E_{{W}_{i}}$$.

By [12, Proposition 2], Assumption 1 guarantees that the cohomologies of the complexes $(W_i,\partial _i)_i$ and $(\widehat{W}_i,\widehat{\partial _i})_i$ are isomorphic. Additionally, the upper diagram in (2.1) is commutative and we have:

2.2 $$\begin{aligned} \widehat{\partial }_i=\widehat{R}_{{W}_{i+1}}{\partial }_iE_{{W}_{i}}. \end{aligned}$$

#### Assumption 2

(*Properties of* $\mathcal{E}_{i}$ and $\mathcal{R}_{i}$) The extension $\mathcal{E}_{i}\,:\,W_i\rightarrow V_i$ and reduction $\mathcal{R}_{i}\,:\,V_i\rightarrow W_i$ are such that

1. $$\mathcal{R}_{i}\mathcal{E}_{i} =\mathrm{Id}_{W_{i}} $$.
2. $$(\mathcal{E}_{i+1}\mathcal{R}_{i+1}-\mathrm{Id}_{V_{i+1}})({{\,\mathrm{Ker}\,}}d_{i+1}) \subset \mathrm{Im}(d_i)$$.
3. $$\mathcal{R}_{i+1} d_i=\partial _i\mathcal{R}_{i}$$ and $$\mathcal{E}_{i+1}\partial _i= d_i \mathcal{E}_{i}$$.

#### Remark 3

*(Isomorphic cohomologies)* Notice that property (B1) is stricter than (A1) since it requires $\mathcal{R}_{i}$ to be a left inverse of $\mathcal{E}_{i}$ on the entire space $W_i$ and not only on ${{\,\mathrm{Ker}\,}}\partial _i$. Accounting for this remark and invoking again [12, Proposition 2], it is easy to see that the cohomologies of $(V_i, d_i)_i$ and $(W_i, \partial _i)_i$ are isomorphic. As noticed above, the latter is, in turn, isomorphic to the cohomology of $(\widehat{W}_i,\widehat{\partial _i})_i$.

#### Lemma 4

(Decomposition of $V_i$) Assume (B1) and let

2.3 $$\begin{aligned} C_i{:}{=}{{\,\mathrm{Ker}\,}}\mathcal{R}_{i}. \end{aligned}$$

Then, we have the following direct decomposition:

2.4 $$\begin{aligned} V_i = \mathcal{E}_{i}W_i \oplus C_i. \end{aligned}$$

Under assumption (B3), this decomposition is compatible with $d_i$, in the sense that

2.5 $$\begin{aligned} d_i \mathcal{E}_{i}W_i \subset \mathcal{E}_{i+1}W_{i+1}\text{ and } d_i C_i \subset C_{i+1}. \end{aligned}$$

#### Proof

By (B1), $\mathcal{R}_{i}$ is surjective and $\mathcal{E}_{i}$ is injective. As a consequence of the latter property, $|W_i|=|\mathcal{E}_{i}W_i|$, where $| \cdot |$ denotes here the dimension of a vector space. By the rank-nullity theorem, we can also write $|C_i|= |V_i| - |\mathrm{Im}\mathcal{R}_{i}| =|V_i|-|W_i|$, where the conclusion follows from the surjectivity of $\mathcal{R}_{i}$. Thus, $|C_i| + |\mathcal{E}_{i}W_i|=|V_i|-|W_i|+|W_i|=|V_i|$, and this gives

$$ V_i=\mathcal{E}_{i}W_i + C_i, $$

thus proving (2.4).

Let us now prove that the sum is direct. To this purpose, let $v\in \mathcal{E}_{i}W_i \cap C_i$. Since $v \in C_i$, $\mathcal{R}_{i} v =0$. Since $v \in \mathcal{E}_{i}W_i$, on the other hand, *v* can be written as $\mathcal{E}_{i}v_w$ for some $v_w \in W_i$, so $\mathcal{R}_{i}\mathcal{E}_{i}v_w=0$. By (B1), $v_w=0$, so $v=\mathcal{E}_{i}0=0$ (since $\mathcal{E}_{i}$ is linear). As a result,

$$ \mathcal{E}_{i}W_i \cap C_i=\{0\}. $$

Now, $\mathcal{R}_{i+1} d_i C_i \overset{(B3)}{=} \partial _i\mathcal{R}_{i} C_i \overset{(2.3)}{=} 0$, giving that $d_i C_i \subset C_{i+1}$. On the other hand, $d_i \mathcal{E}_{i} W_i \overset{(B3)}{=} \mathcal{E}_{i+1} \partial _i W_i$, hence $d_i \mathcal{E}_{i} W_i \subset \mathcal{E}_{i+1} W_{i+1}$. This concludes the proof of (2.5). $\square$

### Construction of a serendipity complex with enhanced regularity

We next construct a new complex $(\widehat{V}_i,\widehat{d}_i)$ with operators

$$ E_{{V}_{i}}\,:\,\widehat{V}_i\rightarrow V_i, \quad \widehat{R}_{{V}_{i}}\,:\,V_i\rightarrow \widehat{V}_i, \quad \widehat{\mathcal{E}}_{i}\,:\,\widehat{W}_i\rightarrow \widehat{V}_i, \quad \widehat{\mathcal{R}}_{i}\,:\,\widehat{V}_i\rightarrow \widehat{W}_i, $$

that verify conditions similar to the ones in Assumptions 1 and 2, so that $(\widehat{V}_i,\widehat{d}_i)$ has the same cohomology as the three other complexes. The construction is illustrated in the following diagram:2.6By Lemma 4, a generic element $v \in V_i$ can be written as $v = \mathcal{E}_{i}v_w + v_c$ with $(v_w, v_c) \in W_i \times C_i$. We introduce the projector $\Pi _{C_i}$ onto $C_i$ such that, for any $v = \mathcal{E}_{i} v_w + v_c$,

2.7 $$\begin{aligned} \Pi _{C_i} v {:}{=}v_c. \end{aligned}$$

Notice that, by definition,

2.8 $$\begin{aligned} \Pi _{C_i} \mathcal{E}_{i} = 0. \end{aligned}$$

In addition, using the compatibility expressed by (2.5),

2.9 $$\begin{aligned} \Pi _{C_{i+1}} d_i v = d_i \Pi _{C_i}v, \end{aligned}$$

as can be checked writing $\Pi _{C_{i+1}} d_i v = \Pi _{C_{i+1}} d_i (\mathcal{E}_{i}v_w + v_c) = \Pi _{C_{i+1}} (d_i \mathcal{E}_{i}v_w + d_i v_c) \overset{(2.5)}{=} d_i v_c \overset{(2.7)}{=} d_i \Pi _{C_i}v$.

#### Definition 5

*(Complex *$(\widehat{V}_i, \widehat{d}_i)$*, extension and reduction operators)* The spaces and differential of the new complex are respectively given by

2.10 $$\begin{aligned} \widehat{V}_i{:}{=}\Big \{\widehat{v} = \big (\widehat{v}_{w},\widehat{v}_{c} \big )\,:\,\, \widehat{v}_{w}\in \widehat{W}_i \text{ and } \widehat{v}_{c}\in C_i \Big \}, \end{aligned}$$

and

2.11 $$\begin{aligned} \widehat{d}_i\widehat{v} {:}{=}(\widehat{\partial }_i\widehat{v}_w, d_i\widehat{v}_c)~\text{for all} ~\widehat{v}= (\widehat{v}_w,\widehat{v}_c) \in \widehat{V}_i. \end{aligned}$$

The operators $\widehat{\mathcal{E}}_{i}\,:\,\widehat{W}_i\rightarrow \widehat{V}_i$, $\widehat{\mathcal{R}}_{i}\,:\,\widehat{V}_i\rightarrow \widehat{W}_i$, $E_{{V}_{i}}\,:\,\widehat{V}_i\rightarrow V_i$, and $\widehat{R}_{{V}_{i}}\,:\,V_i\rightarrow \widehat{V}_i$ relating this new complex to $(\widehat{W}_i,\widehat{\partial }_i)_i$ and $(V_i,d_i)_i$, respectively, are defined as follows:

2.12a $$\begin{aligned} \widehat{\mathcal{E}}_{i} \widehat{v}_w&{:}{=}(\widehat{v}_w,0)&\qquad&\forall \widehat{v}_w \in \widehat{W}_i, \end{aligned}$$

2.12b $$\begin{aligned} \widehat{\mathcal{R}}_{i} \widehat{v}&{:}{=}\widehat{v}_{w}&\qquad&\forall \widehat{v} =(\widehat{v}_{w},\widehat{v}_{c}) \in \widehat{V}_i, \end{aligned}$$

2.12c $$\begin{aligned} E_{{V}_{i}} \widehat{v}&{:}{=}\mathcal{E}_{i}E_{{W}_{i}}\widehat{v}_w + \widehat{v}_c&\qquad&\forall \widehat{v} =(\widehat{v}_{w},\widehat{v}_{c}) \in \widehat{V}_i, \end{aligned}$$

2.12d $$\begin{aligned} \widehat{R}_{{V}_{i}} v&{:}{=}(\widehat{R}_{{W}_{i}}\mathcal{R}_{i}v,\Pi _{C_i} v)&\qquad&\forall v \in V_i. \end{aligned}$$

#### Lemma 6

*(Commutation properties)* Under Assumptions 1 and 2, the operators defined by (2.12) satisfy the following relations:

2.13a $$\begin{aligned} \widehat{R}_{W_i}\mathcal{R}_i&= \widehat{\mathcal{R}}_i\widehat{R}_{V_i},\end{aligned}$$

2.13b $$\begin{aligned} \widehat{\mathcal{E}}_i \widehat{R}_{W_i}&= \widehat{R}_{V_i} \mathcal{E}_i, \end{aligned}$$

2.13c $$\begin{aligned} E_{W_i}\widehat{\mathcal{R}}_i&= \mathcal{R}_i E_{V_i}, \end{aligned}$$

2.13d $$\begin{aligned} \mathcal{E}_i E_{W_i}&= E_{V_i} \widehat{\mathcal{E}}_i, \end{aligned}$$

2.13e $$\begin{aligned} \widehat{\partial _i}\widehat{\mathcal{R}}_{i}&= \widehat{\mathcal{R}}_{i+1}\widehat{d}_i. \end{aligned}$$

#### Proof

$\underline{\mathrm{(i)}\ \textit{Proof}\ \textit{of}\ (2.13a)}$. For all $v \in V_i$, we have

$$ \widehat{\mathcal{R}}_{i}\widehat{R}_{{V}_{i}}v \overset{\mathrm{(2.12d)}}{=}\widehat{\mathcal{R}}_{i}(\widehat{R}_{{W}_{i}}\mathcal{R}_{i}v,\Pi _{C_i} v)\overset{\mathrm{(2.12b)}}{=} \widehat{R}_{{W}_{i}}\mathcal{R}_{i}v. $$

$\underline{\mathrm{(ii)}\ \textit{Proof}\ \textit{of}\ (2.13b)}.$ For all $v_w \in W_i$, it holds

$$\begin{aligned} \widehat{R}_{{V}_{i}}\mathcal{E}_{i}v_w \overset{\mathrm{(2.12d)}}{=} (\widehat{R}_{{W}_{i}}\mathcal{R}_{i}\mathcal{E}_{i}v_w,\Pi _{C_i}\mathcal{E}_{i}v_w) \overset{\mathrm{(B1)},\,\mathrm{(2.8)}}{=} (\widehat{R}_{{W}_{i}}v_w,0) \overset{\mathrm{(2.12a)}}{=}\widehat{\mathcal{E}}_{i}\widehat{R}_{{W}_{i}}v_w. \end{aligned}$$

$\underline{\mathrm{(iii)}\ \textit{Proof} \ \textit{of}\ (2.13c)}.$ For all $\widehat{v} = (\widehat{v}_w,\widehat{v}_c) \in \widehat{V}_i$, we have:

$$\begin{aligned} E_{{W}_{i}}\widehat{\mathcal{R}}_{i}\widehat{v} \overset{(2.12b)}{=} E_{{W}_{i}}\widehat{v}_w \overset{(B1)}{=} \mathcal{R}_{i}\mathcal{E}_{i}E_{{W}_{i}}\widehat{v}_w + \mathcal{R}_{i}\widehat{v}_c = \mathcal{R}_{i}(\mathcal{E}_{i}E_{{W}_{i}}\widehat{v}_w + \widehat{v}_c) \overset{(2.12c)}{=} \mathcal{R}_{i}E_{{V}_{i}}\widehat{v}, \end{aligned}$$

where we have additionally used the fact that $\widehat{v}_c \in C_i$ to add $\mathcal{R}_{i} \widehat{v}_c = 0$ in the right-hand side of the second equality and the linearity of $\mathcal{R}_{i}$ in the third equality. $\underline{\mathrm{(iv)}\ \textit{Proof}\ \textit{of} \ (2.13d).}$ For all $\widehat{v}_w \in \widehat{W}_i$, we can write

$$\begin{aligned} E_{{V}_{i}} \widehat{\mathcal{E}}_{i} \widehat{v}_w \overset{(2.12a)}{=} E_{{V}_{i}}(\widehat{v}_w,0) \overset{(2.12c)}{=} \mathcal{E}_{i}E_{{W}_{i}} \widehat{v}_w . \end{aligned}$$

$\underline{\mathrm{(v)}\ \textit{Proof}\ \textit{of}\ (2.13e).}$ For all ${\widehat{v}} =(\widehat{v}_w,\widehat{v}_c) \in \widehat{V}_i$, we have:

$$\begin{aligned} \widehat{\partial }_i\widehat{\mathcal{R}}_{i} {\widehat{v}} \overset{(2.12b)}{=} \widehat{\partial }_i \widehat{v}_w \overset{(2.12b)}{=} \widehat{\mathcal{R}}_{i+1}(\widehat{\partial }_i \widehat{v}_w, d_i \widehat{v}_c) \overset{(2.11)}{=} \widehat{\mathcal{R}}_{i+1} \widehat{d}_i{\widehat{v}}. \end{aligned}$$

$$\square $$

#### Homological properties

##### Theorem 7

(Homological properties for $(V_i,d_i)_i$and $(\widehat{V}_i,\widehat{d}_i)$) Under Assumptions 1 and 2, the operators $\widehat{R}_{{V}_{i}}$ and $E_{{V}_{i}}$ satisfy the following properties:

2.14a $$\begin{aligned} (\widehat{R}_{{V}_{i}}E_{{V}_{i}})_{|{{\,\mathrm{Ker}\,}}\widehat{d}_i} = \mathrm{Id}_{{{\,\mathrm{Ker}\,}}\widehat{d}_i}, \end{aligned}$$

2.14b $$\begin{aligned} (E_{{V}_{i+1}}\widehat{R}_{{V}_{i+1}}-\mathrm{Id}_{V_{i+1}})({{\,\mathrm{Ker}\,}}d_{i+1}) \subset \mathrm{Im}(d_i), \end{aligned}$$

2.14c $$\begin{aligned} \widehat{R}_{{V}_{i+1}} d_i=\widehat{d}_i\widehat{R}_{{V}_{i}}\text{ and }E_{{V}_{i+1}} \widehat{d}_i=d_iE_{{V}_{i}}. \end{aligned}$$

##### Proof

$\underline{\mathrm{(i)}\ \textit{Proof}\ \textit{ of}\ 2.14a.}$ Let $\widehat{v}=(\widehat{v}_w,\widehat{v}_c) \in {{\,\mathrm{Ker}\,}}\widehat{d}_i$. We have

$$ \begin{aligned} \widehat{R}_{{V}_{i}}E_{{V}_{i}}(\widehat{v}_w,\widehat{v}_c) \overset{\mathrm{(2.12c)}}{=} \widehat{R}_{{V}_{i}}(\mathcal{E}_{i}E_{{W}_{i}}\widehat{v}_w+\widehat{v}_c) \\ \overset{\mathrm{(2.12d)}}{=} \big ( \widehat{R}_{{W}_{i}}\mathcal{R}_{i}\mathcal{E}_{i}E_{{W}_{i}}\widehat{v}_w, \Pi _{C_i} (\mathcal{E}_{i}E_{{W}_{i}}\widehat{v}_w+\widehat{v}_c)\big ) \\ \overset{\mathrm{(B1)},\,\mathrm{(2.7)}}{=} (\widehat{R}_{{W}_{i}}E_{{W}_{i}}\widehat{v}_w,\widehat{v}_c) \\ \overset{\mathrm{(A1)}}{=} (\widehat{v}_w,\widehat{v}_c), \end{aligned} $$

where we have used the linearity of $\mathcal{R}_{i}$ along with $\mathcal{R}_{i} \widehat{v}_c = 0$ (since $\widehat{v}_c \in C_i$) in the second equality, while the use of (A1) in the fourth equality is possible since $\widehat{v}_w \in {{\,\mathrm{Ker}\,}}\widehat{\partial }_i$, as can be checked writing $\widehat{\partial _i} \widehat{v}_w \overset{(2.12b)}{=} \widehat{\partial _i}\widehat{\mathcal{R}}_{i} \widehat{v} \overset{(2.13e)}{=} \widehat{\mathcal{R}}_{i+1}\widehat{d}_i \widehat{v} = 0$, the conclusion being a consequence of $\widehat{v} \in {{\,\mathrm{Ker}\,}}\widehat{d}_i$ and the linearity of $\widehat{\mathcal{R}}_{i+1}$.

$$\underline{\mathrm{(ii)}\ \textit{Proof}\ \textit{of}\ 2.14b.}$$ Let

$$ v \overset{(2.4)}{=} \mathcal{E}_{i+1} v_w + v_c \in {{\,\mathrm{Ker}\,}}d_{i+1}\text{ with }(v_w, v_c) \in W_{i+1} \times C_{i+1}. $$

We write

2.15 $$\begin{aligned}&E_{{V}_{i+1}}\widehat{R}_{{V}_{i+1}} v - v = E_{{V}_{i+1}}\widehat{R}_{{V}_{i+1}}(\mathcal{E}_{i+1} v_w+ v_c)-(\mathcal{E}_{i+1} v_w+ v_c) \\&\overset{\mathrm{(2.12d)}}{=} E_{{V}_{i+1}}\big (\widehat{R}_{{W}_{i+1}} \mathcal{R}_{i+1}(\mathcal{E}_{i+1} v_w+v_c),\Pi _{C_i}(\mathcal{E}_{i+1} v_w+ v_c)\big ) - (\mathcal{E}_{i+1} v_w+ v_c) \\&\overset{\mathrm{(2.7)}}{=} E_{{V}_{i+1}}(\widehat{R}_{{W}_{i+1}} \mathcal{R}_{i+1}\mathcal{E}_{i+1} v_w,v_c) - (\mathcal{E}_{i+1} v_w+ v_c) \\&\overset{\mathrm{(B1)}}{=} E_{{V}_{i+1}}(\widehat{R}_{{W}_{i+1}} v_w, v_c) - (\mathcal{E}_{i+1} v_w+ v_c) \\&\overset{\mathrm{(2.12c)}}{=} \mathcal{E}_{i+1} E_{{W}_{i+1}}\widehat{R}_{{W}_{i+1}} v_w+ v_c- (\mathcal{E}_{i+1} v_w+ v_c) \\&= \mathcal{E}_{i+1} (E_{{W}_{i+1}}\widehat{R}_{{W}_{i+1}} v_w- v_w), \end{aligned}$$

where, in the third equality, we have additionally used the fact that $\mathcal{R}_{i+1} v_c = 0$ since $v_c \in C_{i+1}$. We next notice that $\mathcal{R}_{i+1}v = \mathcal{R}_{i+1}(\mathcal{E}_{i+1}v_w+v_c) = \mathcal{R}_{i+1}\mathcal{E}_{i+1}v_w\overset{(B1)}{=}v_w$. This implies, in turn, $\partial _{i+1}v_w=\partial _{i+1}\mathcal{R}_{i+1}v\overset{(B3)}{=}\mathcal{R}_{i+2}d_{i+1}v=\mathcal{R}_{i+2}0=0$ since $v\in {{\,\mathrm{Ker}\,}}d_{i+1}$ and $\mathcal{R}_{i+2}$ is linear by definition, giving that $v_w\in {{\,\mathrm{Ker}\,}}\partial _i$. We can therefore use Assumption (A2) on $E_{{W}_{i+1}}\widehat{R}_{{W}_{i+1}} v_w- v_w$ in (2.15) to infer the existence of $q\in W_i$ such that

$$\begin{aligned} E_{{V}_{i+1}}\widehat{R}_{{V}_{i+1}} v - v =\mathcal{E}_{i+1} \partial _i q \overset{(B3)}{=} d_i \mathcal{E}_{i} q \in \mathrm{Im}(d_i). \end{aligned}$$

$\underline{\mathrm{(iii)}\ \textit{Proof}\ \textit{ of}\ 2.14c.}$ For all $v \in V_i$, we have

$$\begin{aligned}&\widehat{R}_{{V}_{i+1}} d_i v \overset{\mathrm{(2.12d)}}{=} ( \widehat{R}_{{W}_{i+1}} \mathcal{R}_{i+1} d_i v, \Pi _{C_{i+1}}d_i v ) \\&\overset{\mathrm{(B3)}}{=} ( \widehat{R}_{{W}_{i+1}}\partial _i \mathcal{R}_{i} v ,\Pi _{C_{i+1}}d_i v) \\&\overset{(A3),~\mathrm{(2.9)}}{=} ( \widehat{\partial }_i \widehat{R}_{{W}_{i}} \mathcal{R}_{i}v, d_i \Pi _{C_i} v ) \\&\overset{\mathrm{(2.11)}}{=} \widehat{d}_i (\widehat{R}_{{W}_{i}} \mathcal{R}_{i} v , \Pi _{C_i} v ) \\&\overset{\mathrm{(2.12d)}}{=}\widehat{d}_i \widehat{R}_{{V}_{i}} v. \end{aligned}$$

For all $\widehat{v}=(\widehat{v}_w,\widehat{v}_c) \in \widehat{V}_i$, on the other hand, we have:

$$ \begin{aligned}&E_{{V}_{i+1}} \widehat{d}_i \widehat{v} \overset{\mathrm{(2.11)}}{=} E_{{V}_{i+1}} (\widehat{\partial }_i \widehat{v}_w, d_i\widehat{v}_c ) \\&\overset{\mathrm{(2.12c)}}{=} \mathcal{E}_{i+1} E_{{W}_{i+1}} \widehat{\partial }_i \widehat{v}_w+ d_i \widehat{v}_c \\&\overset{(A3),\,(B3)}{=} d_i \mathcal{E}_{i} E_{{W}_{i}} \widehat{v}_w+ d_i \widehat{v}_c \\&\overset{\mathrm{(2.12c)}}{=} d_i E_{{V}_{i}} (\widehat{v}_w, \widehat{v}_c), \end{aligned} $$

where the conclusion additionally uses the linearity of $d_i$. $\square$

##### Corollary 8

(Isomorphism in cohomology) Under Assumptions 1 and 2, the cohomologies of all the complexes in diagram (2.6) are isomorphic.

##### Proof

Theorem 7 gives all the properties needed to invoke [12, Proposition 2] and prove that the cohomology of the complex $(\widehat{V}_i,\widehat{d}_i)_i$ is isomorphic to that of $(V_i,d_i)_i$. The latter is, on the other hand, isomorphic to both the cohomologies of $(W_i, \partial _i)$ and $(\widehat{W}_i,\widehat{\partial _i})_i$ (see Remark 3). $\square$

#### Polynomial consistency

To close this section, we show that the polynomial consistency properties of the original complexes transfer to $(\widehat{V}_i, \widehat{d}_i)$. This property is typically relevant when considering the local version of the diagram (2.6) on one mesh element.

We denote by $I_{W_i}$, $\widehat{I}_{W_i}$, $I_{V_i}$, and $\widehat{I}_{V_i}$, respectively, the interpolators on the spaces $W_i$, $\widehat{W}_i$, $V_i$, and $\widehat{V}_i$. We assume that their respective domains contain $\mathcal{P}^{k_i}$, a (possibly vector-valued) polynomial space, and that, according to [12, Eq. (2.4)], $\widehat{I}_{W_i}=\widehat{R}_{{W}_{i}} I_{W_i}$ and $\widehat{I}_{V_i}=\widehat{R}_{{V}_{i}} I_{V_i}$.

##### Assumption 9

*(Properties for *$I_{W_i}$*and *$I_{V_i}$) We assume that:

1. $I_{W_i}$ and $\widehat{I}_{W_i}$ meet the following polynomial consistency property:
  2.16 $$\begin{aligned} E_{{W}_{i}} \widehat{I}_{W_i} q_w = E_{{W}_{i}} \widehat{R}_{{W}_{i}} I_{W_i} q_w =I_{W_i}q_w \qquad \forall q_w\in \mathcal{P}^{k_i}, \end{aligned}$$
  expressing the fact that $\widehat{R}_{{W}_{i}}$ is a right inverse of $E_{{W}_{i}}$ on the subspace $I_{W_i} \mathcal{P}^{k_i}$.
2. The interpolator $I_{V_i}$ is consistent with $I_{W_i}$, i.e.,
  2.17 $$\begin{aligned} I_{V_i}-\Pi _{C_i}I_{V_i}=\mathcal{E}_{i}I_{W_i}. \end{aligned}$$

##### Lemma 10

(Polynomial consistency for $(\widehat{V}_i,\widehat{d}_i)$) Under Assumption 9, it holds:

2.18 $$\begin{aligned} E_{{V}_{i}}\widehat{I}_{V_i} q_v = I_{V_i}q_v \qquad \forall q_v \in \mathcal{P}^{k_i}, \end{aligned}$$

i.e., $\widehat{R}_{{V}_{i}}$ is a right inverse of $E_{{V}_{i}}$ on the subspace $I_{V_i} \mathcal{P}^{k_i}$.

##### Proof

By Assumption (C2), we can write for all $q_v\in \mathcal{P}^{k_i}$,

2.19 $$\begin{aligned} I_{V_i} q_v=\mathcal{E}_{i} I_{W_i} q_v + \Pi _{C_i} I_{V_i} q_v. \end{aligned}$$

So we have:

$$ \begin{aligned} E_{{V}_{i}}\widehat{I}_{V_i}q_v&=E_{{V}_{i}}\widehat{R}_{{V}_{i}}I_{V_i}q_v \\&\overset{\mathrm{(2.19)}}{=} E_{{V}_{i}}\widehat{R}_{{V}_{i}}(\mathcal{E}_{i} I_{W_i} q_v + \Pi _{C_i} I_{V_i} q_v) \\&\overset{\mathrm{(2.12d)}}{=}E_{{V}_{i}}(\widehat{R}_{{W}_{i}}\mathcal{R}_{i}\mathcal{E}_{i}I_{W_i}q_v, \Pi _{C_i} I_{V_i} q_v) \\&\overset{\mathrm{(B1)}}{=}E_{{V}_{i}}(\widehat{R}_{{W}_{i}}I_{W_i}q_v, \Pi _{C_i} I_{V_i} q_v) \\&\overset{\mathrm{(2.12c)}}{=}\mathcal{E}_{i}(E_{{W}_{i}}\widehat{R}_{{W}_{i}}I_{W_i}q_v + \Pi _{C_i} I_{V_i} q_v) \\&\overset{\mathrm{(2.16)}}{=}\mathcal{E}_{i}I_{W_i}q_v + \Pi _{C_i} I_{V_i} q_v =I_{V_i}q_v \end{aligned} $$

$$\square $$

## The discrete de Rham complex and its serendipity version

In this section we recall the Discrete De Rham (DDR) complex of [11] and its serendipity version (SDDR) of [12]. These complexes will respectively play the role of $(W_i,\partial _i)_i$ and $(\widehat{W}_i,\widehat{\partial }_i)_i$ in (2.6) for the applications of the following sections. We only give a brief overview of the construction for the sake of conciseness and refer to [11, 12] for additional details.

### Local polynomial spaces and $L^2$-orthogonal projectors

For a polytope $T_d$ embedded in $\mathbb{R}^n$ with $n \ge d$ and an integer $\ell \ge 0$, we denote by $\mathcal{P}^{\ell }(T_d)$ the space spanned by the restriction to $T_d$ of *n*-variate polynomials. Introducing the boldface notation for the space of tangential polynomials $\boldsymbol{\mathcal{P}}^{\ell }(T_d) {:}{=}\mathcal{P}^{\ell }(T_d; \mathbb{R}^d)$ for $d \in \{ 2, 3\}$, the following direct decompositions hold (see, e.g., [1]):

$$ \begin{aligned}&\boldsymbol{\mathcal{P}}^{\ell }(T_2) = \boldsymbol{\mathcal{G}}^{\ell }(T_2) \oplus \boldsymbol{\mathcal{G}}^{\mathrm{c},\ell }(T_2) \\&\mathrm{ with }\boldsymbol{\mathcal{G}}^{\ell }(T_2){:}{=}{\textbf{grad}}_{T_2}\mathcal{P}^{\ell +1}(T_2) \mathrm{ and }\boldsymbol{\mathcal{G}}^{\mathrm{c},\ell }(T_2){:}{=}(\boldsymbol{x}-\boldsymbol{x}_{T_2})^\perp \mathcal{P}^{\ell -1}(T_2), \end{aligned} $$

where ${\textbf{grad}}_{T_2}$ denotes the tangential gradient when $T_2$ is embedded in $\mathbb{R}^3$ and $\boldsymbol{v}^\perp$ is obtained rotating $\boldsymbol{v}$ by $\frac{\pi }{2}$,

$$ \begin{aligned}&\boldsymbol{\mathcal{P}}^{\ell }(T_3) = \boldsymbol{\mathcal{G}}^{\ell }(T_3) \oplus \boldsymbol{\mathcal{G}}^{\mathrm{c},\ell }(T_3) \\&\mathrm{ with }\boldsymbol{\mathcal{G}}^{\ell }(T_3){:}{=}{\textbf{grad}}\mathcal{P}^{\ell +1}(T_3) \mathrm{ and }\boldsymbol{\mathcal{G}}^{\mathrm{c},\ell }(T_3){:}{=}(\boldsymbol{x}-\boldsymbol{x}_{T_3})\times \boldsymbol{\mathcal{P}}^{\ell -1}(T_3), \end{aligned} $$

and, for $$d \in \{ 2, 3 \}$$,

$$ \begin{aligned}&\boldsymbol{\mathcal{P}}^{\ell }(T_d) = \boldsymbol{\mathcal{R}}^{\ell }(T_d) \oplus \boldsymbol{\mathcal{R}}^{\mathrm{c},\ell }(T_d) \\&\mathrm{ with }\boldsymbol{\mathcal{R}}^{\ell }(T_d){:}{=}{\textbf{rot}}_{T_d}\mathcal{P}^{\ell +1}(T_d) \mathrm{ and }\boldsymbol{\mathcal{R}}^{\mathrm{c},\ell }(T_d){:}{=}(\boldsymbol{x}-\boldsymbol{x}_{T_d})\mathcal{P}^{\ell -1}(T_d), \end{aligned} $$

where $${\textbf{rot}}_{T_2} {:}{=}{\textbf{grad}}_{T_2}^\perp $$ and $${\textbf{rot}}_{T_3} {:}{=}{\textbf{curl}}$$.

We extend the above notations to negative exponents $\ell$ by setting all the spaces appearing in the decompositions equal to the trivial vector space. Given a polynomial (sub)space $\mathcal{X}^\ell (T_d)$, the corresponding $L^2$-orthogonal projector is denoted by $\pi _{\mathcal{X},T_d}^\ell$. Boldface font will be used when the elements of $\mathcal{X}^\ell (T_d)$ are vector-valued, and, for $\boldsymbol{\mathcal{X}} \in \{ \boldsymbol{\mathcal{R}}, \boldsymbol{\mathcal{G}} \}$, $\boldsymbol{\pi }_{\boldsymbol{\mathcal{X}},T_d}^{\mathrm{c},\ell }$ denotes the $L^2$-orthogonal projector on $\boldsymbol{\mathcal{X}}^{\mathrm{c},\ell }(T_d)$.

### The two-dimensional discrete de Rham complex

#### Spaces

Given a two-dimensional polygonal mesh $\mathcal{M}_h$, we denote by $\mathcal{M}_{0,h}$, $\mathcal{M}_{1,h}$ and $\mathcal{M}_{2,h}$, respectively, the set of vertices $T_0$, edges $T_1$, and elements $T_2$ of the mesh. Let $k \ge 0$ be a given polynomial degree and, for all $T_2 \in \mathcal{M}_{2,h}$, $n_{T_2}$ and $s_{T_2}$ two integers $\ge -1$ that we collect in the vectors $\boldsymbol{n}= ( n_{T_2} )_{T_2\in \mathcal{M}_{2,h}}$ and $\boldsymbol{s}=( s_{T_2} )_{T_2\in \mathcal{M}_{2,h}}$. The boldface notation is dropped when the values in $\boldsymbol{n}$ and $\boldsymbol{s}$ are all equal.

We define the following discrete counterparts of $H^1(\Omega )$, $\boldsymbol{H}({{\,\mathrm{rot}\,}};\Omega )$, and $L^2(\Omega )$:

$$\begin{aligned} \underline{W}_{{\textbf{grad}},h}^{\boldsymbol{n},k} & {:}{=}\Big \{ \underline{q}_{h}=\big ( (q_{T_2})_{T_2\in \mathcal{M}_{2,h}},(q_{T_1})_{T_1\in \mathcal{M}_{1,h}}, (q_{T_0})_{T_0\in \mathcal{M}_{0,h}} \big )\,:\,\\&\qquad q_{T_2}\in \mathcal{P}^{n_{T_2}}(T_2)\text{ for all }T_2\in \mathcal{M}_{2,h}, \\&\qquad q_{T_1}\in \mathcal{P}^{k-1}(T_1)\text{ for all }T_1\in \mathcal{M}_{1,h}, \\&\qquad q_{T_0}\in \mathbb{R}~\text{for all} ~T_0\in \mathcal{M}_{0,h} \Big \}, \end{aligned} $$

$$\begin{aligned} \underline{\boldsymbol{W}}_{{\textbf{curl}},h}^{\boldsymbol{s},k}{:}&{=}\Big \{ \underline{\boldsymbol{v}}_{w,h}=\big ( (\boldsymbol{v}_{\boldsymbol{\mathcal{R}},T_2},\boldsymbol{v}_{\boldsymbol{\mathcal{R}},T_2}^\mathrm{c})_{T_2\in \mathcal{M}_{2,h}}, (v_{T_1})_{T_1\in \mathcal{M}_{1,h}} \big )\,:\,\\&\qquad \boldsymbol{v}_{\boldsymbol{\mathcal{R}},T_2}\in \boldsymbol{\mathcal{R}}^{k-1}(T_2) \mathrm{ and }\boldsymbol{v}_{\boldsymbol{\mathcal{R}},T_2}^\mathrm{c}\in \boldsymbol{\mathcal{R}}^{\mathrm{c},s_{T_2}}(T_2) \text{ for all }T_2\in \mathcal{M}_{2,h}, \\&\qquad \mathrm{and}~ v_{T_1}\in \mathcal{P}^{k}(T_1) ~\text{for all} ~T_1\in \mathcal{M}_{1,h}\Big \}, \end{aligned} $$

$$ W_{L^2,h}^{k}{:}{=}\mathcal{P}^{k}(\mathcal{M}_{2,h}), $$

where $\mathcal{P}^{k}(\mathcal{M}_{2,h})$ denotes the space of broken polynomials on $\mathcal{M}_{2,h}$ of total degree $\le k$. The restriction of $\underline{W}_{{\textbf{grad}},h}^{\boldsymbol{n},k}$ to an element $T_d$, $d \in \{ 1, 2\}$, is obtained collecting the components on $T_d$ and its boundary and is denoted by $\underline{W}_{{\textbf{grad}},T_d}^{n,k}$. Similar conventions are used for the restriction of the spaces that will appear in the rest of the paper as well as their elements.

#### Interpolators

The interpolators on the two dimensional DDR spaces are defined collecting component-wise $L^2$-projections. Specifically, for smooth enough functions $q: T_2 \rightarrow \mathbb{R}$ and $\boldsymbol{v}: T_2 \rightarrow \mathbb{R}^2$,

$$ \begin{aligned} {{\underline{I}}}_{W,{\textbf{grad}},T_2}^{n_{T_2},k}q&{:}{=}\big ( \pi _{\mathcal{P},T_2}^{n_{T_2}} q, (\pi _{\mathcal{P},T_1}^{k-1} q)_{T_1\in \mathcal{M}_{1,T_2}}, (q(\boldsymbol{x}_{T_0}))_{T_0\in \mathcal{M}_{0,T_2}} \big ), \\ \underline{\boldsymbol{I}}_{\boldsymbol{W},{{\,\mathrm{rot}\,}},T_2}^{s_{T_2},k}\boldsymbol{v}&{:}{=}\big ( \boldsymbol{\pi }_{\boldsymbol{\mathcal{R}},T_2}^{k-1}\boldsymbol{v}, \boldsymbol{\pi }_{\boldsymbol{\mathcal{R}},T_2}^{\mathrm{c},s_{T_2}}\boldsymbol{v}, (\pi _{\mathcal{P},T_1}^{k-1} (\boldsymbol{v}\cdot \boldsymbol{t}_{T_1}))_{T_1\in \mathcal{M}_{1,T_2}} \big ), \end{aligned} $$

and for $W_{L^2,h}^{k}$ the interpolator simply coincides with the $L^2$-orthogonal projector on the broken polynomial space $\mathcal{P}^{k}(\mathcal{M}_{2,h})$.

#### Discrete vector calculus operators

For any edge $T_1\in \mathcal{M}_{1,T_2}$ and any $\underline{q}_{T_1} \in \underline{W}_{{\textbf{grad}},T_1}^{k-1,k}$, the edge gradient $G_{T_1}^k\underline{q}_{T_1}$ is defined as the derivative along $T_1$ of the function $\gamma _{T_1}^{k+1}\underline{q}_{T_1} \in \mathcal{P}^{k+1}(T_1)$ such that $\gamma _{T_1}^{k+1}\underline{q}_{T_1}(\boldsymbol{x}_{T_0}) = q_{T_0}$ for any vertex $T_0$ of $T_1$ of coordinates $\boldsymbol{x}_{T_0}$ and $\pi _{\mathcal{P},T_1}^{k-1} \gamma _{T_1}^{k+1}\underline{q}_{T_1} = q_{T_1}$. We next define the gradient $\boldsymbol{G}_{T_2}^k:\underline{W}_{{\textbf{grad}},T_2}^{k-1,k}\rightarrow \boldsymbol{\mathcal{P}}^{k}(T_2)$ and the scalar two-dimensional potential $\gamma _{T_2}^{k+1}:\underline{W}_{{\textbf{grad}},T_2}^{k-1,k}\rightarrow \mathcal{P}^{k+1}(T_2)$ on $T_2$ such that, for all $\underline{q}_{T_2}\in \underline{W}_{{\textbf{grad}},T_2}^{k-1,k}$,

$$ \int _{T_2}\boldsymbol{G}_{T_2}^k\underline{q}_{T_2}\cdot \boldsymbol{v} = -\int _{T_2} q_{T_2}{{\,\mathrm{div}\,}}_F\boldsymbol{v} + \sum _{T_1\in \mathcal{M}_{1,T_2}}\omega _{T_2T_1}\int _{T_1} \gamma _{T_1}^{k+1}\underline{q}_{T_1}~(\boldsymbol{v}\cdot \boldsymbol{n}_{T_2T_1}) \quad \forall \boldsymbol{v}\in \boldsymbol{\mathcal{P}}^{k}(T_2), $$

3.1 $$\begin{aligned} \int _{T_2}\gamma _{T_2}^{k+1}\underline{q}_{T_2}{{\,\mathrm{div}\,}}_{T_2}\boldsymbol{v} = -\int _{T_2}\boldsymbol{G}_{T_2}^k\underline{q}_{T_2}\cdot \boldsymbol{v} + \sum _{T_1\in \mathcal{M}_{1,T_2}}\omega _{T_2T_1}\int _{T_1} \gamma _{T_1}^{k+1}\underline{q}_{T_1}~(\boldsymbol{v}\cdot \boldsymbol{n}_{T_2T_1}) \\ \forall \boldsymbol{v}\in \boldsymbol{\mathcal{R}}^{\mathrm{c},k+2}(T_2), \end{aligned}$$

where $\boldsymbol{n}_{T_2T_1}$ is a unit normal vector to $T_1$ lying in the plane of $T_2$ and $\omega _{T_2 T_1}$ the orientation of $T_1$ relative to $T_2$ such that $\omega _{T_2T_1} \boldsymbol{n}_{T_2T_1}$ points out of $T_2$.

The two-dimensional scalar rotor $C_{T_2}^{k}:\underline{\boldsymbol{W}}_{{\textbf{curl}},T_2}^{k,k}\rightarrow \mathcal{P}^{k}(T_2)$ and the corresponding vector potential $\boldsymbol{\gamma }_{\mathrm{t},T_2}^k:\underline{\boldsymbol{W}}_{{\textbf{curl}},T_2}^{k,k}\rightarrow \boldsymbol{\mathcal{P}}^{k}(T_2)$ (which can be interpreted as a tangential component when $T_2$ is the face of a polyhedron) are such that, for all $\underline{\boldsymbol{v}}_{T_2}\in \underline{\boldsymbol{W}}_{{\textbf{curl}},T_2}^{k,k}$,

$$ \int _{T_2}C_{T_2}^{k}\underline{\boldsymbol{v}}_{T_2}~r = \int _{T_2}\boldsymbol{v}_{\boldsymbol{\mathcal{R}},{T_2}}\cdot {\textbf{rot}}_{T_2} r - \sum _{T_1\in \mathcal{M}_{1,T_2}}\omega _{T_2T_1}\int _{T_1} v_{T_1}~r \qquad \forall r\in \mathcal{P}^{k}(T_2), $$

3.2 $$\begin{aligned} \int _{T_2}\boldsymbol{\gamma }_{\mathrm{t},T_2}^k\underline{\boldsymbol{v}}_{T_2}\cdot ({\textbf{rot}}_{T_2} r + \boldsymbol{w}) = \int _{T_2}C_{T_2}^{k}\underline{\boldsymbol{v}}_{T_2}~r + \sum _{T_1\in \mathcal{M}_{1,T_2}}\omega _{T_2T_1}\int _{T_1} v_{T_1}~r + \int _{T_2}\boldsymbol{v}_{\boldsymbol{\mathcal{R}},{T_2}}^\mathrm{c}\cdot \boldsymbol{w} \\ \forall (r,\boldsymbol{w})\in \mathcal{P}^{k+1}(T_2)\times \boldsymbol{\mathcal{R}}^{\mathrm{c},k}(T_2). \end{aligned}$$

We will also need the two-dimensional vector rotor $\boldsymbol{C}^k_{T_2}:\underline{\boldsymbol{W}}_{{\textbf{curl}},T_2}^{k,k}\rightarrow \boldsymbol{\mathcal{P}}^{k}(T_2)$ such that

3.3 $$\begin{aligned} \int _{T_2} \boldsymbol{C}^k_{T_2} \underline{\boldsymbol{v}}_{T_2} \cdot \boldsymbol{w} = \int _{T_2} v_{T_2} {{\,\mathrm{rot}\,}}\boldsymbol{w} + \sum _{T_1\in \mathcal{M}_{1,T_2}} \omega _{T_2T_1} \int _{T_1} (\boldsymbol{v}_{T_1} \cdot \boldsymbol{n}_{T_2T_1}) (\boldsymbol{w} \cdot \boldsymbol{t}_{T_1}) \qquad \forall \boldsymbol{w} \in \boldsymbol{\mathcal{P}}^{k}(T_2). \end{aligned}$$

#### DDR complex

The two-dimensional DDR complex of degree *k* reads3.4where the discrete global gradient $\underline{\boldsymbol{\partial }}_{{\textbf{grad}},h}^k$ and curl $\partial _{{{\,\mathrm{rot}\,}},h}^k$ are such that, for all $(\underline{q}_{h}, \underline{\boldsymbol{v}}_{h}) \in \underline{W}_{{\textbf{grad}},h}^{k-1,k} \times \underline{\boldsymbol{W}}_{{\textbf{curl}},h}^{k,k}$,

$$\begin{aligned} \underline{\boldsymbol{\partial }}_{{\textbf{grad}},h}^k \underline{q}_{h} {:}{=}\big ( (\boldsymbol{\pi }_{\boldsymbol{\mathcal{R}},T_2}^{k-1}\boldsymbol{G}_{T_2}^k\underline{q}_{T_2},\boldsymbol{\pi }_{\boldsymbol{\mathcal{R}},T_2}^{\mathrm{c},k}\boldsymbol{G}_{T_2}^k\underline{q}_{T_2})_{T_2\in \mathcal{M}_{2,h}}, ( G_{T_1}^kq_{T_1} )_{T_1\in \mathcal{M}_{1,h}} \big ), \\ ( \partial _{{{\,\mathrm{rot}\,}},h}^k \underline{\boldsymbol{v}}_{h} )_{| T_2} {:}{=}C_{T_2}^{k}\underline{\boldsymbol{v}}_{T_2}~ \text{for all}~ T_2\in \mathcal{M}_{2,h}. \end{aligned} $$

### The three-dimensional discrete de Rham complex

#### Spaces

Let us now consider a three-dimensional mesh $\mathcal{M}_h$, with $\mathcal{M}_{0,h}$, $\mathcal{M}_{1,h}$, $\mathcal{M}_{2,h}$, and $\mathcal{M}_{3,h}$ denoting, respectively, the set of vertices $T_0$, edges $T_1$, faces $T_2$, and elements $T_3$. Given four vectors of integers $\ge -1$ $\boldsymbol{m}{:}{=}(m_{T_3})_{T_3\in \mathcal{M}_{3,h}}$, $\boldsymbol{n}{:}{=}(n_{T_2})_{T_2\in \mathcal{M}_{2,h}}$, $\boldsymbol{p}{:}{=}(p_{T_3})_{T_3\in \mathcal{M}_{3,h}}$, and $\boldsymbol{s}{:}{=}(s_{T_2})_{T_2\in \mathcal{M}_{3,h}}$, we define the following discrete counterparts of $H^1(\Omega )$, $\boldsymbol{H}({\textbf{curl}};\Omega )$, $\boldsymbol{H}({{\,\mathrm{div}\,}};\Omega )$, and $L^2(\Omega )$:

$$\begin{aligned} \underline{W}_{{\textbf{grad}},h}^{\boldsymbol{m},\boldsymbol{n},k}& {:}{=}\Big \{ \underline{q}_{w,h}=\big ( (q_{T_3})_{T_3\in \mathcal{M}_{3,h}},(q_{T_2})_{T_2\in \mathcal{M}_{2,h}},(q_{T_1})_{T_1\in \mathcal{M}_{1,h}}, (q_{T_0})_{T_0\in \mathcal{M}_{0,h}} \big )\,:\,\\&\qquad q_{T_3}\in \mathcal{P}^{m_{T_3}}(T_3) \text{ for all }T_3 \in \mathcal{M}_{3,h}, \\&\qquad q_{T_2}\in \mathcal{P}^{n_{T_2}}(T_2) \text{ for all }T_2 \in \mathcal{M}_{2,h}, \\&\qquad q_{T_1}\in \mathcal{P}^{k-1}(T_1)\text{ for all }T_1\in \mathcal{M}_{1,h}, \\&\qquad \mathrm{and}~ q_{T_0}\in \mathbb{R}~\text{for all} ~T_0\in \mathcal{M}_{0,h} \Big \}, \end{aligned} $$

$$\begin{aligned} \underline{\boldsymbol{W}}_{{\textbf{curl}},h}^{\boldsymbol{p},\boldsymbol{s},k} & {:}{=}\Big \{ \underline{\boldsymbol{v}}_{w,h}=\big ( (\boldsymbol{v}_{\boldsymbol{\mathcal{R}},T_3},\boldsymbol{v}_{\boldsymbol{\mathcal{R}},T_3}^\mathrm{c})_{T_3\in \mathcal{M}_{3,h}}, (\boldsymbol{v}_{\boldsymbol{\mathcal{R}},T_2},\boldsymbol{v}_{\boldsymbol{\mathcal{R}},T_2}^\mathrm{c})_{T_2\in \mathcal{M}_{2,h}}, (v_{T_1})_{T_1\in \mathcal{M}_{1,h}} \big )\,:\,\\&\qquad \boldsymbol{v}_{\boldsymbol{\mathcal{R}},T_3}\in \boldsymbol{\mathcal{R}}^{k-1}(T_3)\mathrm{ and }\boldsymbol{v}_{\boldsymbol{\mathcal{R}},T_3}^\mathrm{c}\in \boldsymbol{\mathcal{R}}^{\mathrm{c},p_{T_3}}(T_3)\text{ for all }T_3\in \mathcal{M}_{3,h}, \\&\qquad \boldsymbol{v}_{\boldsymbol{\mathcal{R}},T_2}\in \boldsymbol{\mathcal{R}}^{k-1}(T_2)\mathrm{ and }\boldsymbol{v}_{\boldsymbol{\mathcal{R}},T_2}^\mathrm{c}\in \boldsymbol{\mathcal{R}}^{\mathrm{c},s_{T_2}}(T_2) \text{ for all }T_2\in \mathcal{M}_{2,h}, \\&\qquad \mathrm{and}~ v_{T_1}\in \mathcal{P}^{k}(T_1)~ \text{for all} ~T_1\in \mathcal{M}_{1,h}\Big \}, \end{aligned} $$

$$\begin{aligned} \underline{\boldsymbol{W}}_{{{\,\mathrm{div}\,}},h}^{k}{:}&{=}\Big \{ \underline{\boldsymbol{w}}_{w,h}=\big ((\boldsymbol{w}_{\boldsymbol{\mathcal{G}},T_3},\boldsymbol{w}_{\boldsymbol{\mathcal{G}},T_3}^\mathrm{c})_{T_3\in \mathcal{M}_{3,h}}, (w_{T_2})_{T_2\in \mathcal{M}_{2,h}}\big )\,:\,\\&\qquad \boldsymbol{w}_{\boldsymbol{\mathcal{G}},T_3}\in \boldsymbol{\mathcal{G}}^{k-1}(T_3) \mathrm{ and }\boldsymbol{w}_{\boldsymbol{\mathcal{G}},T_3}^\mathrm{c}\in \boldsymbol{\mathcal{G}}^{\mathrm{c},k}(T_3)\text{ for all }T_3\in \mathcal{M}_{3,h}, \\&\qquad \mathrm{and}~ w_{T_2}\in \mathcal{P}^{k}(T_2) ~\text{for all} ~T_2\in \mathcal{M}_{2,T_3} \Big \}, \end{aligned} $$

and

$$ W_{L^2,h}^{k} {:}{=}\mathcal{P}^{k}(\mathcal{M}_{3,h}). $$

When the values in $\boldsymbol{m}$, $\boldsymbol{n}$, $\boldsymbol{p}$ and $\boldsymbol{s}$ are all equal, where we drop the boldface notation. With a little abuse in notation, for the discrete gradient operator defined by (3.9) below as well as for the tail space $W_{L^2,h}^{k}$, we use the same symbols as for the DDR2d sequence: all ambiguity will be removed by the context.

#### Discrete vector calculus operators

The element gradient $\boldsymbol{G}_{T_3}^k:\underline{W}_{{\textbf{grad}},T_3}^{k-1,k-1,k}\rightarrow \boldsymbol{\mathcal{P}}^{k}(T_3)$, the element curl $\boldsymbol{C}_{T_3}^{k}:\underline{\boldsymbol{W}}_{{\textbf{curl}},T_3}^{k,k,k}\rightarrow \boldsymbol{\mathcal{P}}^{k}(T_3)$, and the element divergence $D_{T_3}^{k}:\underline{\boldsymbol{W}}_{{{\,\mathrm{div}\,}},T_3}^{k}\rightarrow \mathcal{P}^{k}(T_3)$ are respectively defined such that, for all $\underline{q}_{T_3}\in \underline{W}_{{\textbf{grad}},T_3}^{k-1,k-1,k}$, all $\underline{\boldsymbol{v}}_{T_3}\in \underline{\boldsymbol{W}}_{{\textbf{curl}},T_3}^{k,k,k}$, and all $\underline{\boldsymbol{w}}_{T_3}\in \underline{\boldsymbol{W}}_{{{\,\mathrm{div}\,}},T_3}^{k}$,

3.5 $$\begin{aligned} \int _{T_3}\boldsymbol{G}_{T_3}^k\underline{q}_{T_3}\cdot \boldsymbol{v} = -\int _{T_3} q_{T_3}{{\,\mathrm{div}\,}}\boldsymbol{v} + \sum _{T_2\in \mathcal{M}_{2,T_3}}\omega _{T_3T_2}\int _{T_2}\gamma _{T_2}^{k+1}\underline{q}_{T_2}~(\boldsymbol{v}\cdot \boldsymbol{n}_{T_2}) \quad \forall \boldsymbol{v}\in \boldsymbol{\mathcal{P}}^{k}(T_3), \end{aligned}$$

3.6 $$\begin{aligned} \int _{T_3}\boldsymbol{C}_{T_3}^{k}\underline{\boldsymbol{v}}_{T_3}\cdot \boldsymbol{z} = \int _{T_3}\boldsymbol{v}_{\boldsymbol{\mathcal{R}},T_3}\cdot {\textbf{curl}}\boldsymbol{z} + \sum _{T_2\in \mathcal{M}_{2,T_3}}\omega _{T_3T_2}\int _{T_2}\boldsymbol{\gamma }_{\mathrm{t},T_2}^k\underline{\boldsymbol{v}}_{T_2}\cdot (\boldsymbol{z}\times \boldsymbol{n}_{T_2}) \\ \forall \boldsymbol{z}\in \boldsymbol{\mathcal{P}}^{k}(T_3), \end{aligned}$$

3.7 $$\begin{aligned} \int _{T_3}D_{T_3}^{k}\underline{\boldsymbol{w}}_{T_3}~q = -\int _{T_3}\boldsymbol{w}_{\boldsymbol{\mathcal{G}},T_3}\cdot {\textbf{grad}}q + \sum _{T_2\in \mathcal{M}_{2,T_3}}\omega _{T_3T_2}\int _{T_2} w_{T_2}~q \qquad \forall q\in \mathcal{P}^{k}(T_3), \end{aligned}$$

where $\boldsymbol{n}_{T_2}$ is a unit normal vector to $T_2$ and $\omega _{T_3T_2}$ is the orientation of $T_2$ relative to $T_3$ such that $\omega _{T_3 T_2} \boldsymbol{n}_{T_2}$ points out of $T_3$.

#### Interpolators

The interpolators on the three-dimensional DDR spaces are defined such that, for smooth enough functions $q: T_3 \rightarrow \mathbb{R}$, $\boldsymbol{v}: T_3 \rightarrow \mathbb{R}^3$, and $\boldsymbol{w}: T_3 \rightarrow \mathbb{R}^3$,

$$\begin{aligned} {{\underline{I}}}_{W,{\textbf{grad}},T_3}^{m_{T_3},n_{T_2},k}q&{:}{=}\big ( \pi _{\mathcal{P},T_3}^{m_{T_3}}q, (\pi _{\mathcal{P},T_2}^{n_{T_2}} q)_{T_2\in \mathcal{M}_{2,T_3}}, (\pi _{\mathcal{P},T_1}^{k-1} q)_{T_1\in \mathcal{M}_{1,T_3}}, (q(\boldsymbol{x}_{T_0}))_{T_0\in \mathcal{M}_{0,T_3}} \big ), \\ \underline{\boldsymbol{I}}_{\boldsymbol{W},{\textbf{curl}},T_3}^{p_{t_3},s_{T_2},k}\boldsymbol{v}&{:}{=}\big ( \boldsymbol{\pi }_{\boldsymbol{\mathcal{R}},T_3}^{k-1}\boldsymbol{v}, \boldsymbol{\pi }_{\boldsymbol{\mathcal{R}},T_3}^{\mathrm{c},p_{T_3}}\boldsymbol{v}, (\boldsymbol{\pi }_{\boldsymbol{\mathcal{R}},T_2}^{k-1}\boldsymbol{v}, \boldsymbol{\pi }_{\boldsymbol{\mathcal{R}},T_2}^{\mathrm{c},s_{T_2}}\boldsymbol{v})_{T_2\in \mathcal{M}_{2,T_3}}, (\pi _{\mathcal{P},T_1}^{k-1} \boldsymbol{v}\cdot \boldsymbol{t}_{T_1})_{T_1\in \mathcal{M}_{1,T_2}} \big ), \\ \underline{\boldsymbol{I}}_{\boldsymbol{W},{{\,\mathrm{div}\,}},T_3}^{k}\boldsymbol{w}&{:}{=}\big ( \boldsymbol{\pi }_{\boldsymbol{\mathcal{G}},T_3}^{k-1}\boldsymbol{w}, \boldsymbol{\pi }_{\boldsymbol{\mathcal{G}},T_3}^{\mathrm{c},k}\boldsymbol{w}, (\pi _{\mathcal{P},T_2}^{k}\boldsymbol{w}_{|T_2}\cdot \boldsymbol{n}_{T_1})_{T_2\in \mathcal{M}_{2,T_3}} \big ). \end{aligned} $$

#### DDR complex

The global three-dimensional DDR complex of degree *k* is3.8where the operators $\underline{\boldsymbol{\partial }}_{{\textbf{grad}},h}^k$, $\underline{\boldsymbol{\partial }}_{{\textbf{curl}},h}^{k}$ and $\partial _{{{\,\mathrm{div}\,}},h}^k$ are obtained projecting the element and face operators onto the component spaces: For all $(\underline{q}_{h},\underline{\boldsymbol{v}}_{h},\underline{\boldsymbol{w}}_{h})\in \underline{W}_{{\textbf{grad}},h}^{k-1,k-1,k}\times \underline{\boldsymbol{W}}_{{\textbf{curl}},h}^{k,k,k}\times \underline{\boldsymbol{W}}_{{{\,\mathrm{div}\,}},h}^{k}$,

3.9 $$\begin{aligned} \underline{\boldsymbol{\partial }}_{{\textbf{grad}},h}^k \underline{q}_{h} &{:}{=} \big ((\boldsymbol{\pi }_{\boldsymbol{\mathcal{R}},T_3}^{k-1}\boldsymbol{G}_{T_3}^k\underline{q}_{T_3},\boldsymbol{\pi }_{\boldsymbol{\mathcal{R}},T_3}^{\mathrm{c},k}\boldsymbol{G}_{T_3}^k\underline{q}_{T_3})_{T_3\in \mathcal{M}_{3,h}}, \\&( \boldsymbol{\pi }_{\boldsymbol{\mathcal{R}},T_2}^{k-1}\boldsymbol{G}_{T_2}^k\underline{q}_{T_2},\boldsymbol{\pi }_{\boldsymbol{\mathcal{R}},T_2}^{\mathrm{c},k}\boldsymbol{G}_{T_2}^k\underline{q}_{T_2} )_{T_2\in \mathcal{M}_{2,h}}, \\&( G_{T_1}^kq_{T_1} )_{T_1\in \mathcal{M}_{1,h}} \big ), \\ \underline{\boldsymbol{\partial }}_{{\textbf{curl}},h}^{k}\underline{\boldsymbol{v}}_{h} &{:}{=}\big ( (\boldsymbol{\pi }_{\boldsymbol{\mathcal{G}},T_3}^{k-1}\boldsymbol{C}_{T_3}^{k}\underline{\boldsymbol{v}}_{T_3},\boldsymbol{\pi }_{\boldsymbol{\mathcal{G}},T_3}^{\mathrm{c},k}\boldsymbol{C}_{T_3}^{k}\underline{\boldsymbol{v}}_{T_3})_{T_3\in \mathcal{M}_{3,h}}, ( C_{T_2}^{k}\underline{\boldsymbol{v}}_{T_2} )_{T_2\in \mathcal{M}_{2,h}} \big ), \\ &( \partial _{{{\,\mathrm{div}\,}},h}^k\underline{\boldsymbol{w}}_{h} )_{| T_3} {:}{=}D_{T_3}^{k}\underline{\boldsymbol{w}}_{T_3}\text{ for all }T_3\in \mathcal{M}_{3,h}.\end{aligned}$$

### Serendipity spaces

We now introduce the two- and three-dimensional Serendipity Discrete de Rham (SDDR) complexes that will play the role of $(\widehat{W}_i,\widehat{\partial }_i)_i$ in the applications considered in Sects. 4 and 5 below.

For each $T_d \in \mathcal{M}_{d,h}$, $d \in \{2, 3\}$, we select $\eta _{T_d}\ge 2$ faces/edges that are not pairwise aligned and such that ${T_d}$ lies entirely on one side of the plane/line spanned by each of those faces/edges. The exact requirements are detailed in [12, Assumption 12]. We then set

$$ \ell _{T_d} {:}{=}k + 1 - \eta _{T_d}. $$

These integers are collected in the vector $\boldsymbol{\ell }_d {:}{=}( \ell _{T_d} )_{T_d \in \mathcal{M}_{d,h}}$. The serendipity version of the spaces in (3.4) and (3.8) are, respectively,

3.10 $$\begin{aligned} \underline{\widehat{W}}_{{\textbf{grad}},h}^{k}&{:}{=}\underline{W}_{{\textbf{grad}},h}^{\boldsymbol{\ell }_2,k},&\qquad \underline{\widehat{\boldsymbol{W}}}_{{{\,\mathrm{rot}\,}},h}^k&{:}{=}\underline{\boldsymbol{W}}_{{{\,\mathrm{rot}\,}},h}^{\boldsymbol{\ell }_2+1,k}, \\ \underline{\widehat{W}}_{{\textbf{grad}},h}^{k}&{:}{=}\underline{W}_{{\textbf{grad}},h}^{\boldsymbol{\ell }_3,\boldsymbol{\ell }_2,k},&\qquad \underline{\widehat{\boldsymbol{W}}}_{{\textbf{curl}},h}^k&{:}{=}\underline{\boldsymbol{W}}_{{\textbf{curl}},h}^{\boldsymbol{\ell }_3 +1,\boldsymbol{\ell }_2 +1,k}. \end{aligned}$$

In these spaces, the degree of certain polynomial components inside faces and elements for which $\eta _{T_d} > 2$ is lower than in the non-serendipity spaces defined in Sects. 3.2.1 and 3.3.1, the more so the larger $\eta _{T_d}$.

#### Remark 11

(Algorithm to compute $\eta _{T_d}$) To select a serendipity edge/face, we test each one: First, we ensure that the cell lies on only one side of the newly selected edge/face by checking that the dot product between the edge/face normal and the vector from each cell vertex to the edge/face midpoint is negative. Next, we verify that the newly selected edge/face is not aligned with (i.e. contained within the tangent line or plane of) any previously selected edge/face by confirming that the dot product of the vector from the edge/face midpoint to the midpoint of each already selected edge/face exceeds a specific threshold, which is proportional to the cell’s skewness.

Since $\eta _{T_d}$ depends on the geometry of the element, the dimension of the local spaces can no longer be determined solely by the number of faces/edges/vertices of an element, and must be tracked during the construction. However, this is a mild inconvenience, and not fundamentally different from the fact that two elements with different numbers of edges/faces have different local spaces.

### Extension and reduction maps between the two-dimensional DDR and SDDR complexes

Following [12, Section 5.3], for a polygon $T_2$ it is possible to define serendipity gradient and rotor operators $\boldsymbol{S}_{{\textbf{grad}},T_2}^k:\underline{\widehat{W}}_{{\textbf{grad}},T_2}^{k}\rightarrow \boldsymbol{\mathcal{P}}^{k}(T_2)$ and $\boldsymbol{S}_{{{\,\mathrm{rot}\,}},T_2}^k:\underline{\widehat{\boldsymbol{W}}}_{{{\,\mathrm{rot}\,}},T_2}^k\rightarrow \boldsymbol{\mathcal{P}}^{k}(T_2)$ that satisfy the following properties:

$$ \begin{aligned} \boldsymbol{S}_{{\textbf{grad}},T_2}^k{\underline{{\widehat{I}}}}_{W,{\textbf{grad}},T_2}^{k} q = {\textbf{grad}}_{T_2}q \qquad \forall q \in \mathcal{P}^{k+1}(T_2), \\ \boldsymbol{S}_{{{\,\mathrm{rot}\,}},T_2}^k\underline{\widehat{\boldsymbol{I}}}_{\boldsymbol{W},{{\,\mathrm{rot}\,}},T_2}^{k} \boldsymbol{v}=\boldsymbol{v} \qquad \forall \boldsymbol{v} \in \boldsymbol{\mathcal{P}}^{k}(T_2), \end{aligned} $$

where ${\underline{{\widehat{I}}}}_{W,{\textbf{grad}},T_2}^{k}$ and $\underline{\widehat{\boldsymbol{I}}}_{\boldsymbol{W},{{\,\mathrm{rot}\,}},T_2}^{k}$ are defined, for smooth enough functions $q: T_2 \rightarrow \mathbb{R}$ and $\boldsymbol{v}: T_2 \rightarrow \mathbb{R}^2$, as follows:

$$ \begin{aligned} {\underline{{\widehat{I}}}}_{W,{\textbf{grad}},T_2}^{k}q&{:}{=}\big ( \pi _{\mathcal{P},T_2}^{\ell _{T_2}} q, (\pi _{\mathcal{P},T_1}^{k-1} q)_{T_1\in \mathcal{M}_{1,T_2}}, (q(\boldsymbol{x}_{T_0}))_{T_0\in \mathcal{M}_{0,T_2}} \big ), \\ \underline{\widehat{\boldsymbol{I}}}_{\boldsymbol{W},{{\,\mathrm{rot}\,}},T_2}^{k}\boldsymbol{v}&{:}{=}\big ( \boldsymbol{\pi }_{\boldsymbol{\mathcal{R}},T_2}^{k-1}\boldsymbol{v}, \boldsymbol{\pi }_{\boldsymbol{\mathcal{R}},T_2}^{\mathrm{c},\ell _{T_2}+1}\boldsymbol{v}, (\pi _{\mathcal{P},T_1}^{k-1} (\boldsymbol{v}\cdot \boldsymbol{t}_{T_1}))_{T_1\in \mathcal{M}_{1,T_2}} \big ), \end{aligned} $$

The role of the serendipity operators is to reconstruct polynomial fields inside $T_2$ from the polynomial components of the serendipity spaces.

In order to define two-dimensional extension maps, we need an operator $E_{\mathcal{P} ,T_2}^{k-1}:\underline{\widehat{W}}_{{\textbf{grad}},T_2}^{k}\rightarrow \mathcal{P}^{k-1}(T_2)$ that satisfies a formal integration by parts with the serendipity gradient: For all $\boldsymbol{w}\in \boldsymbol{\mathcal{R}}^{\mathrm{c},k}(T_2)$,

$$ \int _FE_{\mathcal{P} ,T_2}^{k-1}\underline{\widehat{q}}_{T_2}{{\,\mathrm{div}\,}}_{T_2}\boldsymbol{w} = - \int _{T_2}\boldsymbol{S}_{{\textbf{grad}},T_2}^k\underline{\widehat{q}}_{T_2}\cdot \boldsymbol{w} + \sum _{T_i\in \mathcal{M}_{1,T_2}}\omega _{T_2 T_1} \int _{T_1} \widehat{q}_{T_1}~(\boldsymbol{w}\cdot \boldsymbol{n}_{T_2T_1}). $$

The extension operators $\underline{E}_{W,{\textbf{grad}},h}:\underline{\widehat{W}}_{{\textbf{grad}},h}^{k}\rightarrow \underline{W}_{{\textbf{grad}},h}^{k-1,k}$ and $\underline{\boldsymbol{E}}_{\boldsymbol{W},{{\,\mathrm{rot}\,}},h}:\underline{\widehat{\boldsymbol{W}}}_{{{\,\mathrm{rot}\,}},h}^k\rightarrow \underline{\boldsymbol{W}}_{{{\,\mathrm{rot}\,}},h}^{k,k}$ are defined by

3.11 $$\begin{aligned} \underline{E}_{W,{\textbf{grad}},h}\underline{\widehat{q}}_{h} {:}{=}\big ( (E_{\mathcal{P} ,T_2}^{k-1}\underline{\widehat{q}}_{T_2})_{T_2 \in \mathcal{M}_{2,h}}, (\widehat{q}_{T_1})_{T_1 \in \mathcal{M}_{1,h}}, (\widehat{q}_{T_0})_{T_0 \in \mathcal{M}_{0,h}} \big ) \qquad \forall \underline{\widehat{q}}_{h}\in \underline{\widehat{W}}_{{\textbf{grad}},h}^{k}, \end{aligned}$$

3.12 $$\begin{aligned} \underline{\boldsymbol{E}}_{\boldsymbol{W},{{\,\mathrm{rot}\,}},h}\underline{\widehat{\boldsymbol{v}}}_{h} {:}{=}\big ( (\widehat{\boldsymbol{v}}_{\boldsymbol{\mathcal{R}},T_2}, \boldsymbol{\pi }_{\boldsymbol{\mathcal{R}},T_2}^{\mathrm{c},k}\boldsymbol{S}_{{{\,\mathrm{rot}\,}},T_2}^k \underline{\widehat{\boldsymbol{v}}}_{T_2})_{T_2\in \mathcal{M}_{2,h}}, (\widehat{v}_{T_1})_{T_1\in \mathcal{M}_{1,h}} \big ) \qquad \forall \underline{\widehat{\boldsymbol{v}}}_{h}\in \underline{\widehat{\boldsymbol{W}}}_{{{\,\mathrm{rot}\,}},h}^k, \end{aligned}$$

while the reduction operators $\underline{\widehat{R}}_{\boldsymbol{W},{\textbf{grad}},h}:\underline{W}_{{\textbf{grad}},h}^{k-1,k}\rightarrow \underline{\widehat{W}}_{{\textbf{grad}},h}^{k}$ and $\underline{\widehat{\boldsymbol{R}}}_{\boldsymbol{W},{{\,\mathrm{rot}\,}},h}:\underline{\boldsymbol{W}}_{{{\,\mathrm{rot}\,}},h}^{k,k} \rightarrow \underline{\widehat{\boldsymbol{W}}}_{{{\,\mathrm{rot}\,}},h}^k$ are such that

3.13 $$\begin{aligned} \underline{\widehat{R}}_{\boldsymbol{W},{\textbf{grad}},h} \underline{q}_{h} {:}{=}\big ( (\pi _{\mathcal{P},T_2}^{\ell _{T_2}}q_{T_2})_{T_2 \in \mathcal{M}_{2,h}}, (q_{T_1})_{T_1 \in \mathcal{M}_{1,h}}, (q_{T_0})_{T_0 \in \mathcal{M}_{0,h}} \big ) \qquad \forall \underline{q}_{h}\in \underline{W}_{{\textbf{grad}},h}^{k-1,k}, \end{aligned}$$

3.14 $$\begin{aligned} \underline{\widehat{\boldsymbol{R}}}_{\boldsymbol{W},{{\,\mathrm{rot}\,}},h}\underline{\boldsymbol{v}}_{h} {:}{=}\big ( (\boldsymbol{v}_{\boldsymbol{\mathcal{R}},T_2}, \boldsymbol{\pi }_{\boldsymbol{\mathcal{R}},T_2}^{\mathrm{c},\ell _{T_2}+1}\boldsymbol{v}_{\boldsymbol{\mathcal{R}},T_2}^\mathrm{c})_{T_2 \in \mathcal{M}_{2,h}}, (v_{T_1})_{T_1\in \mathcal{M}_{1,T_2}} \big ) \qquad \forall \underline{\boldsymbol{v}}_{h}\in \underline{\boldsymbol{W}}_{{{\,\mathrm{rot}\,}},h}^{k,k}. \end{aligned}$$

The complexes $(W_i,\partial _i)_i$ and $(\widehat{W}_i,\widehat{\partial }_i)_i$ along with the corresponding extension and reduction maps that will be used in the application of Sect. 4 are summarized in the following diagram:3.15where $\underline{\widehat{\boldsymbol{\partial }}}_{{\textbf{grad}},h}^{k}$ and $\widehat{\partial }_{{{\,\mathrm{rot}\,}},h}^{k}$ are given by (2.2).

### Extension and reduction maps between the three-dimensional DDR and SDDR complexes

Now, taking a polyhedron $T_3$ and following again [12, Section 5.4], it is possible to define serendipity gradient and curl operators $\boldsymbol{S}_{{\textbf{grad}},T_3}^k:\underline{\widehat{W}}_{{\textbf{grad}},T_3}^{k}\rightarrow \mathcal{P}^{k}(T_3)$ and $\boldsymbol{S}_{{\textbf{curl}},T_3}^k:\underline{\widehat{\boldsymbol{W}}}_{{\textbf{curl}},T_3}^k\rightarrow \mathcal{P}^{k}(T_3)$ that satisfy the following properties:

$$ \begin{aligned} \boldsymbol{S}_{{\textbf{grad}},T_3}^k{\underline{{\widehat{I}}}}_{W,{\textbf{grad}},T_3}^{k} q={\textbf{grad}}_{T_3}q \qquad \forall q \in \mathcal{P}^{k+1}(T_3), \\ \boldsymbol{S}_{{{\,\mathrm{rot}\,}},T_3}^k\underline{\widehat{\boldsymbol{I}}}_{\boldsymbol{W},{\textbf{curl}},T_3}^{k} \boldsymbol{v}=\boldsymbol{v} \qquad \forall \boldsymbol{v} \in \boldsymbol{\mathcal{P}}^{k}(T_3), \end{aligned} $$

where ${\underline{{\widehat{I}}}}_{W,{\textbf{grad}},T_3}^{k}$ and $\underline{\widehat{\boldsymbol{I}}}_{\boldsymbol{W},{\textbf{curl}},T_3}^{k}$ are defined, for smooth enough functions $q: T_3 \rightarrow \mathbb{R}$, $\boldsymbol{v}: T_3 \rightarrow \mathbb{R}^3$, and $\boldsymbol{w}: T_3 \rightarrow \mathbb{R}^3$, as follows:

$$\begin{aligned} {\underline{{\widehat{I}}}}_{W,{\textbf{grad}},T_3}^{k}q&{:}{=}\big ( \pi _{\mathcal{P},T_3}^{\ell _{T_3}}q, (\pi _{\mathcal{P},T_2}^{\ell _{T_2}} q)_{T_2\in \mathcal{M}_{2,T_3}}, (\pi _{\mathcal{P},T_1}^{k-1} q)_{T_1\in \mathcal{M}_{1,T_3}}, (q(\boldsymbol{x}_{T_0}))_{T_0\in \mathcal{M}_{0,T_3}} \big ), \\ \underline{\widehat{\boldsymbol{I}}}_{\boldsymbol{W},{\textbf{curl}},T_3}^{k}\boldsymbol{v}&{:}{=}\big ( \boldsymbol{\pi }_{\boldsymbol{\mathcal{R}},T_3}^{k-1}\boldsymbol{v}, \boldsymbol{\pi }_{\boldsymbol{\mathcal{R}},T_3}^{\mathrm{c},\ell _{T_3}+1}\boldsymbol{v}, (\boldsymbol{\pi }_{\boldsymbol{\mathcal{R}},T_2}^{k-1}\boldsymbol{v}, \boldsymbol{\pi }_{\boldsymbol{\mathcal{R}},T_2}^{\mathrm{c},\ell _{T_2}+1}\boldsymbol{v})_{T_2\in \mathcal{M}_{2,T_3}}, (\pi _{\mathcal{P},T_1}^{k-1} \boldsymbol{v}\cdot \boldsymbol{t}_{T_1})_{T_1\in \mathcal{M}_{1,T_2}} \big ) ,\\ \underline{\boldsymbol{I}}_{\boldsymbol{W},{{\,\mathrm{div}\,}},T_3}^{k}\boldsymbol{w}&{:}{=}\big ( \boldsymbol{\pi }_{\boldsymbol{\mathcal{G}},T_3}^{k-1}\boldsymbol{w}, \boldsymbol{\pi }_{\boldsymbol{\mathcal{G}},T_3}^{\mathrm{c},k}\boldsymbol{w}, (\pi _{\mathcal{P},T_2}^{k}\boldsymbol{w}_{|T_2}\cdot \boldsymbol{n}_{T_1})_{T_2\in \mathcal{M}_{2,T_3}} \big ). \end{aligned} $$

We also define $E_{\mathcal{P} ,T}^{k-1}:\underline{\widehat{W}}_{{\textbf{grad}},T_3}^{k}\rightarrow \mathcal{P}^{k-1}(T_3)$ such that, for all $\boldsymbol{w}\in \boldsymbol{\mathcal{R}}^{\mathrm{c},k}(T_3)$,

$$ \int _{T_3}E_{\mathcal{P} ,T}^{k-1}\underline{\widehat{q}}_{T_3}{{\,\mathrm{div}\,}}\boldsymbol{w} = - \int _{T_3}\boldsymbol{S}_{{\textbf{grad}},T_3}^k\underline{\widehat{q}}_T\cdot \boldsymbol{w} + \sum _{T_2\in \mathcal{M}_{{T_2}\in {T_3}}}\omega _{T_3T_2}\int _{T_2} \widehat{q}_{T_2}~(\boldsymbol{w}\cdot \boldsymbol{n}_{T_2}), $$

$\widehat{R}^{\ell _{T_3}}_{\mathcal{P} ,T_3}:\underline{W}_{{\textbf{grad}},T_3}^{k-1,k-1,k}\rightarrow \mathcal{P}^{\ell _{T_3}}(T_3)$, such that, for all $\boldsymbol{w}\in \boldsymbol{\mathcal{R}}^{\mathrm{c},\ell _{T_3}+1}(T_3)$,

$$\begin{aligned} \int _{T_3}\widehat{R}^{\ell _{T_3}}_{\mathcal{P} ,T_3}\underline{q}_{T_3}{{\,\mathrm{div}\,}}\boldsymbol{w} =-\int _{T_3}\boldsymbol{G}_{T_3}^k\underline{q}_{T_3}\cdot \boldsymbol{w} \\ + \sum _{T_2\in \mathcal{M}_{2,T_3}}\omega _{T_3 T_2}\int _{T_2} \gamma _{T_2}^{k+1}\underline{E}_{W,{\textbf{grad}},T_2}\underline{\widehat{R}}_{\boldsymbol{W},{\textbf{grad}},T_2}\underline{q}_{T_2}~(\boldsymbol{w}\cdot \boldsymbol{n}_{T_2}), \end{aligned}$$

and $\widehat{\boldsymbol{R}}^{k-1}_{\boldsymbol{\mathcal{R}},T_3}:\underline{\widehat{\boldsymbol{W}}}_{{\textbf{curl}},T_3}^k\rightarrow \boldsymbol{\mathcal{R}}^{k-1}(T_3)$ such that, for all $\boldsymbol{w}\in \boldsymbol{\mathcal{G}}^{\mathrm{c},k}(T_3)$,

$$\begin{aligned} \int _{T_3} \widehat{\boldsymbol{R}}^{k-1}_{\boldsymbol{\mathcal{R}},T_3}\underline{\boldsymbol{v}}_{T_3}\cdot {\textbf{curl}}\boldsymbol{w} = \int _{T_3}\boldsymbol{C}_{T_3}^{k}\underline{\boldsymbol{v}}_{T_3}\cdot \boldsymbol{w} \\ - \sum _{T_2\in \mathcal{M}_{2,T_3}}\omega _{T_3T_2}\int _{T_2}\boldsymbol{\gamma }_{\mathrm{t},T_2}^k\underline{\boldsymbol{E}}_{\boldsymbol{W},{\textbf{curl}},T_2}\underline{\widehat{\boldsymbol{R}}}_{\boldsymbol{W},{\textbf{curl}},T_2}\underline{\boldsymbol{v}}_{T_2}\cdot (\boldsymbol{w}\times \boldsymbol{n}_{T_2}). \end{aligned}$$

where $\gamma _{T_2}^{k+1}$, $\boldsymbol{\gamma }_{\mathrm{t},T_2}^k$, $\boldsymbol{G}_{T_3}^k$, and $\boldsymbol{C}_{T_3}^{k}$, are respectively defined by (3.1), (3.2), (3.5), and (3.6).

The extension operators $\underline{E}_{W,{\textbf{grad}},h}:\underline{\widehat{W}}_{{\textbf{grad}},h}^{k}\rightarrow \underline{W}_{{\textbf{grad}},h}^{k-1,k-1,k}$ and $\underline{\boldsymbol{E}}_{\boldsymbol{W},{\textbf{curl}},h}:\underline{\widehat{\boldsymbol{W}}}_{{\textbf{curl}},h}^k\rightarrow \underline{\boldsymbol{W}}_{{\textbf{curl}},h}^{k,k,k}$ are such that, for all $\underline{\widehat{q}}_{h}\in \underline{\widehat{W}}_{{\textbf{grad}},h}^{k}$ and all $\underline{\widehat{\boldsymbol{v}}}_{h}\in \underline{\widehat{\boldsymbol{W}}}_{{\textbf{curl}},h}^k$,

$$ \begin{aligned} \underline{E}_{W,{\textbf{grad}},h}\underline{\widehat{q}}_{h} {:}{=}\big ( (E_{\mathcal{P} ,T_3}^{k-1}\underline{\widehat{q}}_{T_3})_{T_3\in \mathcal{M}_{3,h}}, (E_{\mathcal{P} ,T_2}^{k-1}\underline{\widehat{q}}_{T_2})_{T_2\in \mathcal{M}_{2,h}}, (\widehat{q}_{T_1})_{T_1 \in \mathcal{M}_{1,h}}, (\widehat{q}_{T_0})_{T_0 \in \mathcal{M}_{0,h}} \big ), \\ \underline{\boldsymbol{E}}_{\boldsymbol{W},{\textbf{curl}},h}\underline{\widehat{\boldsymbol{v}}}_{h} {:}{=}\big ( (\widehat{\boldsymbol{v}}_{\boldsymbol{\mathcal{R}},T_3}, \boldsymbol{\pi }_{\boldsymbol{\mathcal{R}},T_3}^{\mathrm{c},k}\boldsymbol{S}_{{\textbf{curl}},T_3}^k\underline{\widehat{\boldsymbol{v}}}_{T_3})_{T_3\in \mathcal{M}_{3,h}}, (\widehat{\boldsymbol{v}}_{\boldsymbol{\mathcal{R}},T_2}, \boldsymbol{\pi }_{\boldsymbol{\mathcal{R}},T_2}^{\mathrm{c},k}\boldsymbol{S}_{{\textbf{curl}},T_2}^k\underline{\widehat{\boldsymbol{v}}}_{T_2})_{T_2\in \mathcal{M}_{2,h}}, (\widehat{v}_{T_1})_{T_1\in \mathcal{M}_{1,h}} \big ), \end{aligned} $$

while the reduction operators are $\underline{\widehat{R}}_{\boldsymbol{W},{\textbf{grad}},h}:\underline{W}_{{\textbf{grad}},h}^{k-1,k-1,k}\rightarrow \underline{\widehat{W}}_{{\textbf{grad}},h}^{k}$ and $\underline{\widehat{\boldsymbol{R}}}_{\boldsymbol{W},{\textbf{curl}},h}:\underline{\boldsymbol{W}}_{{\textbf{curl}},h}^{k,k,k}\rightarrow \underline{\widehat{\boldsymbol{W}}}_{{\textbf{curl}},h}^k$ such that, for all $\underline{q}_{h}\in \underline{W}_{{\textbf{grad}},h}^{k-1,k-1,k}$ and all $\underline{\boldsymbol{v}}_{h}\in \underline{\boldsymbol{W}}_{{\textbf{curl}},h}^{k,k,k}$,

$$ \begin{aligned} \underline{\widehat{R}}_{\boldsymbol{W},{\textbf{grad}},h}\underline{q}_{h} {:}{=}\big ( (\widehat{R}^{\ell _{T_3}}_{\mathcal{P} ,T_3}\underline{q}_{T_3})_{T_3\in \mathcal{M}_{3,h}}, (\pi _{\mathcal{P},T_2}^{\ell _{T_2}}q_{T_2})_{T_2\in \mathcal{M}_{2,h}}, (q_{T_1})_{T_1 \in \mathcal{M}_{1,h}}, (q_{T_0})_{T_0 \in \mathcal{M}_{0,h}} \big ), \\ \underline{\widehat{\boldsymbol{R}}}_{\boldsymbol{W},{\textbf{curl}},h}\underline{\boldsymbol{v}}_{h} {:}{=}\big ( (\widehat{\boldsymbol{R}}^{k-1}_{\boldsymbol{\mathcal{R}},T_3}\underline{\boldsymbol{v}}_{T_3}, \boldsymbol{\pi }_{\boldsymbol{\mathcal{R}},T_3}^{\mathrm{c},\ell _{T_3}+1}\boldsymbol{v}_{\boldsymbol{\mathcal{R}},{T_3}}^\mathrm{c})_{T_3\in \mathcal{M}_{3,h}}, (\boldsymbol{v}_{\boldsymbol{\mathcal{R}},T_2},\boldsymbol{\pi }_{\boldsymbol{\mathcal{R}},T_2}^{\mathrm{c},\ell _{T_2}+1}\boldsymbol{v}_{\boldsymbol{\mathcal{R}},T_2}^\mathrm{c})_{T_2\in \mathcal{M}_{2,h}}, (v_{T_1} \big )_{T_1\in \mathcal{M}_{1,h}}). \end{aligned} $$

The complexes $(W_i,\partial _i)_i$ and $(\widehat{W}_i,\widehat{\partial }_i)_i$ for the application of Sect. 5 along with the corresponding extension and reduction maps are summarized in the following diagram:3.16where $\underline{\widehat{\boldsymbol{\partial }}}_{{\textbf{grad}},h}^{k}$ and $\underline{\widehat{\boldsymbol{\partial }}}_{{\textbf{curl}},h}^{k}$ are given by (2.2).

### Cohomology of the serendipity DDR complexes

We recall the following result from [12] (see, in particular, Lemmas 22 and 26 therein).

#### Lemma 12

(Cohomology of the DDR and SDDR complexes) The two- and three-dimensional DDR and SDDR complexes, together with their extension and reduction operators, satisfy Assumption 1. In particular, this implies that both the cohomologies of the SDDR and DDR complexes are isomorphic to the cohomology of the corresponding continuous de Rham complex.

### Polynomial consistency

By [12, Eq. (2.4)], the interpolators ${\underline{{\widehat{I}}}}_{W,{\textbf{grad}},T_2}^{k}$, $\underline{\widehat{\boldsymbol{I}}}_{\boldsymbol{W},{{\,\mathrm{rot}\,}},T_2}^{k}$, ${\underline{{\widehat{I}}}}_{W,{\textbf{grad}},T_3}^{k}$, and $\underline{\widehat{\boldsymbol{I}}}_{\boldsymbol{W},{\textbf{curl}},T_d}^{k}$ respectively on the spaces $\underline{\widehat{W}}_{{\textbf{grad}},T_2}^{k}$, $\underline{\widehat{\boldsymbol{W}}}_{{{\,\mathrm{rot}\,}},T_2}^k$, $\underline{\widehat{W}}_{{\textbf{grad}},T_3}^{k}$, and $\underline{\widehat{\boldsymbol{W}}}_{{\textbf{curl}},T_3}^k$ are such that, for smooth enough $q: T_d \rightarrow \mathbb{R}$ and $\boldsymbol{v}: T_d \rightarrow \mathbb{R}^d$, $d\in \{2,3\}$,

$$ \begin{aligned} {\underline{{\widehat{I}}}}_{W,{\textbf{grad}},T_2}^{k}q&{:}{=}\underline{\widehat{R}}_{\boldsymbol{W},{\textbf{grad}},T_2}{{\underline{I}}}_{W,{\textbf{grad}},T_2}^{n_{T_2},k}q,\\ \underline{\widehat{\boldsymbol{I}}}_{\boldsymbol{W},{{\,\mathrm{rot}\,}},T_2}^{k}\boldsymbol{v}&{:}{=}\underline{\widehat{\boldsymbol{R}}}_{\boldsymbol{W},{{\,\mathrm{rot}\,}},T_2}\underline{\boldsymbol{I}}_{\boldsymbol{W},{{\,\mathrm{rot}\,}},T_2}^{s_{T_2},k}\boldsymbol{v},\\ {\underline{{\widehat{I}}}}_{W,{\textbf{grad}},T_3}^{k}q&{:}{=}\underline{\widehat{R}}_{\boldsymbol{W},{\textbf{grad}},T_3}{{\underline{I}}}_{W,{\textbf{grad}},T_3}^{m_{T_3},n_{T_2},k}q,\\ \underline{\widehat{\boldsymbol{I}}}_{\boldsymbol{W},{\textbf{curl}},T_3}^{k}\boldsymbol{v}&{:}{=}\underline{\widehat{\boldsymbol{R}}}_{\boldsymbol{W},{\textbf{curl}},T_3}\underline{\boldsymbol{I}}_{\boldsymbol{W},{\textbf{curl}},T_3}^{p_{T_3},s_{T_2},k}\boldsymbol{v}. \end{aligned} $$

#### Lemma 13

(Polynomial consistency of the SDDR complexes) The interpolators in Sects. 3.2.2 and 3.3.3 and their serendipity version verify the polynomial consistency property:

$$ \begin{aligned} \underline{E}_{W,{\textbf{grad}},T_2}{\underline{{\widehat{I}}}}_{W,{\textbf{grad}},T_2}^{k}q&= {{\underline{I}}}_{W,{\textbf{grad}},T_2}^{n_{T_2},k}q&\qquad&\forall q \in \mathcal{P}^{k+1}(T_2), \\ \underline{\boldsymbol{E}}_{\boldsymbol{W},{{\,\mathrm{rot}\,}},T_2}\underline{\widehat{\boldsymbol{I}}}_{\boldsymbol{W},{{\,\mathrm{rot}\,}},T_2}^{k}\boldsymbol{v}&=\underline{\boldsymbol{I}}_{\boldsymbol{W},{{\,\mathrm{rot}\,}},T_2}^{s_{T_2},k}\boldsymbol{v}&\qquad&\forall \boldsymbol{v}\in \boldsymbol{\mathcal{P}}^{k}(T_2), \\ \underline{E}_{W,{\textbf{grad}},T_3}{\underline{{\widehat{I}}}}_{W,{\textbf{grad}},T_3}^{k}q&= {{\underline{I}}}_{W,{\textbf{grad}},T_3}^{n_{T_2},k}q&\qquad&\forall q \in \mathcal{P}^{k+1}(T_3), \\ \underline{\boldsymbol{E}}_{\boldsymbol{W},{\textbf{curl}},T_3}\underline{\widehat{\boldsymbol{I}}}_{\boldsymbol{W},{\textbf{curl}},T_3}^{k}\boldsymbol{v}&=\underline{\boldsymbol{I}}_{\boldsymbol{W},{\textbf{curl}},T_3}^{p_{T_3},s_{T_2},k}\boldsymbol{v}&\qquad&\forall \boldsymbol{v}\in \boldsymbol{\mathcal{P}}^{k}(T_3). \end{aligned} $$

## A serendipity rot-rot complex

We now turn to the first application of the general construction considering the smoother variant (1.2) of the two-dimensional de Rham complex. Diagram (2.6) specialized to the present case becomes4.1The top horizontal portion of the above diagram corresponds to (3.15). In the rest of this section we will provide a precise definition of the other spaces and operators that appear in it and, using the abstract framework of Sect. 2, show that all the complexes involved have isomorphic cohomologies.

### Discrete rot-rot complex

A discrete counterpart of the complex (1.2) was developed in [8]. We briefly recall its construction here. We define the discrete head $H^1(\Omega )$, $\boldsymbol{H}({\textbf{rot}}{{\,\mathrm{rot}\,}};\Omega )$, and tail $H^1(\Omega )$ spaces as follows:

$$ \underline{V}_{{\textbf{grad}},h}^k {:}{=}\underline{W}_{{\textbf{grad}},h}^{k-1,k},\quad \underline{\boldsymbol{V}}_{{{\,\mathrm{rot}\,}},h}^k {:}{=}\underline{\boldsymbol{W}}_{{{\,\mathrm{rot}\,}},h}^{k,k}\times \left( \times _{T_1\in \mathcal{M}_{1,h}}\mathcal{P}^{k-1}(T_1)\times \mathbb{R}^{\mathcal{M}_{0,h}} \right) ,\quad \underline{V}_{H^1,h}^k {:}{=}\underline{W}_{{\textbf{grad}},h}^{k,k}. $$

The discrete gradient and rotor are respectively such that, for all $\underline{q}_h\in \underline{V}_{{\textbf{grad}},h}^k$ and all $\underline{\boldsymbol{v}}_h=\big (\underline{\boldsymbol{v}}_{w,h}, {\underline{v}}_{\mathrm{c},h} \big )\in \underline{\boldsymbol{V}}_{{{\,\mathrm{rot}\,}},h}^k$,

4.2 $$\begin{aligned} \underline{\boldsymbol{d}}_{{\textbf{grad}},{h}}^k~\underline{q}_h&{:}{=}\big ( \underline{\boldsymbol{\partial }}_{{\textbf{grad}},h}^k\underline{q}_h,\underline{0} \big ), \end{aligned}$$

4.3 $$\begin{aligned} \underline{d}_{{{\,\mathrm{rot}\,}},h}^k\underline{\boldsymbol{v}}_h&{:}{=}\big ( \partial _{{{\,\mathrm{rot}\,}},h}^k\underline{\boldsymbol{v}}_{w,h}, \underline{v}_{\mathrm{c},h} \big ). \end{aligned}$$

The discrete counterpart of (1.2) is then given by:4.4

### Extension and reduction maps between the two-dimensional DDR and rot-rot complexes

In order to apply the construction of Definition 5 to define and characterize a serendipity version of this complex, we need extension and reduction maps between the two-dimensional DDR complex (3.4) and the discrete rot-rot complex (4.4). Noticing that

$$ \underline{V}_{H^1,h}^k=W_{L^2,h}^{k}\times \left( \times _{T_1\in \mathcal{M}_{1,h}}\mathcal{P}^{k-1}(T_1)\times \mathbb{R}^{\mathcal{M}_{0,h}} \right) , $$

the spaces $\underline{\boldsymbol{W}}_{{{\,\mathrm{rot}\,}},h}^{k,k}$ and $W_{L^2,h}^{k}$ inject respectively into $\underline{\boldsymbol{V}}_{{{\,\mathrm{rot}\,}},h}^k$ and $\underline{V}_{H^1,h}^k$ trough the extension map such that, for all $\underline{\boldsymbol{v}}_{w,h}\in \underline{\boldsymbol{W}}_{{{\,\mathrm{rot}\,}},h}^{k,k}$ and all $q_h\in W_{L^2,h}^{k}$,

4.5 $$\begin{aligned} \underline{\boldsymbol{{\mathcal{E}}}}_{{{\,\mathrm{rot}\,}},h}^k\underline{\boldsymbol{v}}_{w,h} {:}{=}\big ( \underline{\boldsymbol{v}}_{w,h}, \underline{0}\big ) \text{ and } \underline{{\mathcal{E}}}_{H^1,h}^kq_h {:}{=}\big ( q_h,\underline{0}\big ). \end{aligned}$$

We also define the reduction map such that, for all $\underline{\boldsymbol{v}}_h=(\underline{\boldsymbol{v}}_{w,h},\underline{v}_{\mathrm{c},h})\in \underline{\boldsymbol{V}}_{{{\,\mathrm{rot}\,}},h}^k$ and all $\underline{q}_h=(q_h,\underline{q}_{\mathrm{c},h})\in \underline{V}_{H^1,h}^k$,

4.6 $$\begin{aligned} \underline{\boldsymbol{{\mathcal{R}}}}_{{{\,\mathrm{rot}\,}},h}^k\underline{\boldsymbol{v}}_h {:}{=}\underline{\boldsymbol{v}}_{w,h} \text{ and } \mathcal{R}_{H^1,h}^k\underline{q}_h {:}{=}q_h. \end{aligned}$$

The decomposition of Lemma 4 clearly holds by definition, so we have

$$ \underline{\boldsymbol{V}}_{{{\,\mathrm{rot}\,}},h}^k=\underline{\boldsymbol{{\mathcal{E}}}}_{{{\,\mathrm{rot}\,}},h}^k\underline{\boldsymbol{W}}_{{{\,\mathrm{rot}\,}},h}^{k,k} \oplus {{\,\mathrm{Ker}\,}}\underline{\boldsymbol{{\mathcal{R}}}}_{{{\,\mathrm{rot}\,}},h}^k\text{ and }\underline{V}_{H^1,h}^k=\underline{{\mathcal{E}}}_{H^1,h}^k W_{L^2,h}^{k}\oplus {{\,\mathrm{Ker}\,}}\mathcal{R}_{H^1,h}^k. $$

#### Theorem 14

(Properties of the extension and reduction maps between the DDR2d and rot-rot complexes) The maps defined by (4.5) and (4.6) satisfy Assumption 2, i.e.,

1. For all $\underline{\boldsymbol{v}}_{w,h}\in \underline{\boldsymbol{W}}_{{{\,\mathrm{rot}\,}},h}^{k,k}$ and all $q_h\in W_{L^2,h}^{k}$,
  4.7 $$\begin{aligned} \underline{\boldsymbol{{\mathcal{R}}}}_{{{\,\mathrm{rot}\,}},h}^k\underline{\boldsymbol{{\mathcal{E}}}}_{{{\,\mathrm{rot}\,}},h}^k\underline{\boldsymbol{v}}_{w,h} = \underline{\boldsymbol{v}}_{w,h} \text{ and } \mathcal{R}_{H^1,h}^k\underline{{\mathcal{E}}}_{H^1,h}^kq_h = q_h. \end{aligned}$$
2. For all $$\underline{\boldsymbol{v}}_h=(\underline{\boldsymbol{v}}_{w,h},\underline{v}_{\mathrm{c},h})\in {{\,\mathrm{Ker}\,}}\underline{d}_{{{\,\mathrm{rot}\,}},h}^k$$,
  4.8 $$\begin{aligned} \underline{\boldsymbol{{\mathcal{E}}}}_{{{\,\mathrm{rot}\,}},h}^k\underline{\boldsymbol{{\mathcal{R}}}}_{{{\,\mathrm{rot}\,}},h}^k\underline{\boldsymbol{v}}_h-\underline{\boldsymbol{v}}_h\in \mathrm{Im}(\underline{\boldsymbol{d}}_{{\textbf{grad}},{h}}^k). \end{aligned}$$
3. For all $\underline{q}_h\in \underline{V}_{{\textbf{grad}},h}^k$, all $\underline{\boldsymbol{v}}_h\in \underline{\boldsymbol{V}}_{{{\,\mathrm{rot}\,}},h}^k$, and all $\underline{\boldsymbol{v}}_{w,h}\in \underline{\boldsymbol{W}}_{{{\,\mathrm{rot}\,}},h}^{k,k}$, it holds
  4.9 $$\begin{aligned} \underline{\boldsymbol{{\mathcal{R}}}}_{{{\,\mathrm{rot}\,}},h}^k\underline{\boldsymbol{d}}_{{\textbf{grad}},{h}}^k\underline{q}_h&= \underline{\boldsymbol{\partial }}_{{\textbf{grad}},h}^k \underline{q}_h,&\qquad \underline{\boldsymbol{{\mathcal{E}}}}_{{{\,\mathrm{rot}\,}},h}^k\underline{\boldsymbol{\partial }}_{{\textbf{grad}},h}^k \underline{q}_h&= \underline{\boldsymbol{d}}_{{\textbf{grad}},{h}}^k\underline{q}_h, \end{aligned}$$
  4.10 $$\begin{aligned} \mathcal{R}_{H^1,h}^k\underline{d}_{{{\,\mathrm{rot}\,}},h}^k \underline{\boldsymbol{v}}_h&= \partial _{{{\,\mathrm{rot}\,}},h}^k \underline{\boldsymbol{{\mathcal{R}}}}_{{{\,\mathrm{rot}\,}},h}^k\underline{\boldsymbol{v}}_h,&\qquad \underline{{\mathcal{E}}}_{H^1,h}^k \partial _{{{\,\mathrm{rot}\,}},h}^k \underline{\boldsymbol{v}}_{w,h}&= \underline{d}_{{{\,\mathrm{rot}\,}},h}^k\underline{\boldsymbol{{\mathcal{E}}}}_{{{\,\mathrm{rot}\,}},h}^k\underline{\boldsymbol{v}}_{w,h}. \end{aligned}$$

It then follows from Remark 3 that the two-dimensional DDR complex (3.4) and the rot-rot complex (4.4) have isomorphic cohomologies.

#### Proof

$\underline{\mathrm{(i)}\, \textit{Proof} \, \textit{ of } \, (4.7).}$ For all $\underline{\boldsymbol{v}}_{w,h}\in \underline{\boldsymbol{W}}_{{{\,\mathrm{rot}\,}},h}^{k,k}$, $\underline{\boldsymbol{{\mathcal{R}}}}_{{{\,\mathrm{rot}\,}},h}^k\underline{\boldsymbol{{\mathcal{E}}}}_{{{\,\mathrm{rot}\,}},h}^k\underline{\boldsymbol{v}}_{w,h}\overset{(4.5)}{=}\underline{\boldsymbol{{\mathcal{R}}}}_{{{\,\mathrm{rot}\,}},h}^k(\underline{\boldsymbol{v}}_{w,h},\underline{0})\overset{(4.6)}{=}\underline{\boldsymbol{v}}_{w,h}$ and, for all $q_h\in W_{L^2,h}^{k}$, $\mathcal{R}_{H^1,h}^k\underline{{\mathcal{E}}}_{H^1,h}^kq_h\overset{(4.5)}{=}\mathcal{R}_{H^1,h}^k(q_h,\underline{0})\overset{(4.6)}{=}q_h$.

$\underline{\mathrm{(ii)} \, \textit{Proof} \, \textit{ of } \, (4.8).}$ Let $\underline{\boldsymbol{v}}_h\in {{\,\mathrm{Ker}\,}}\underline{d}_{{{\,\mathrm{rot}\,}},h}^k$. Using the definition (4.3) of $\underline{d}_{{{\,\mathrm{rot}\,}},h}^k$, we obtain that $\underline{\boldsymbol{v}}_h=(\underline{\boldsymbol{v}}_{w,h},\underline{0})$, so $\underline{\boldsymbol{{\mathcal{E}}}}_{{{\,\mathrm{rot}\,}},h}^k\underline{\boldsymbol{{\mathcal{R}}}}_{{{\,\mathrm{rot}\,}},h}^k\underline{\boldsymbol{v}}_h-\underline{\boldsymbol{v}}_h=\underline{\boldsymbol{0}}=\underline{\boldsymbol{d}}_{{\textbf{grad}},{h}}^k\underline{0}.$

$\underline{\mathrm{(iii)} \, \textit{Proof} \, \textit{ of } \, (4.9).}$ For all $\underline{q}_h\in \underline{V}_{{\textbf{grad}},h}^k$, we have $\underline{\boldsymbol{{\mathcal{R}}}}_{{{\,\mathrm{rot}\,}},h}^k\underline{\boldsymbol{d}}_{{\textbf{grad}},{h}}^k\underline{q}_h \overset{(4.2)}{=} \underline{\boldsymbol{{\mathcal{R}}}}_{{{\,\mathrm{rot}\,}},h}^k(\underline{\boldsymbol{\partial }}_{{\textbf{grad}},h}^k\underline{q}_h,\underline{0}) \overset{(4.6)}{=} \underline{\boldsymbol{\partial }}_{{\textbf{grad}},h}^k\underline{q}_h$ and $\underline{\boldsymbol{{\mathcal{E}}}}_{{{\,\mathrm{rot}\,}},h}^k\underline{\boldsymbol{\partial }}_{{\textbf{grad}},h}^k\underline{q}_h \overset{(4.5)}{=} (\underline{\boldsymbol{\partial }}_{{\textbf{grad}},h}^k\underline{q}_h,\underline{0}) \overset{(4.2)}{=} \underline{\boldsymbol{d}}_{{\textbf{grad}},{h}}^k\underline{q}_h$.

$$\underline{\mathrm{(vi)} \, \textit{Proof } \, \textit{ of } \, (4.10).}$$ For all $$\underline{\boldsymbol{v}}_h=(\underline{\boldsymbol{v}}_{w,h},\underline{v}_{\mathrm{c},h})\in \underline{\boldsymbol{V}}_{{{\,\mathrm{rot}\,}},h}^k$$,

$$ \mathcal{R}_{H^1,h}^k\underline{d}_{{{\,\mathrm{rot}\,}},h}^k(\underline{\boldsymbol{v}}_{w,h},\underline{v}_{\mathrm{c},h}) \overset{(4.3)}{=} \mathcal{R}_{H^1,h}^k(\partial _{{{\,\mathrm{rot}\,}},h}^k\underline{\boldsymbol{v}}_{w,h},\underline{v}_{\mathrm{c},h}) \overset{(4.6)}{=} \partial _{{{\,\mathrm{rot}\,}},h}^k\underline{\boldsymbol{v}}_{w,h} \overset{(4.6)}{=} \partial _{{{\,\mathrm{rot}\,}},h}^k\underline{\boldsymbol{{\mathcal{R}}}}_{{{\,\mathrm{rot}\,}},h}^k\underline{\boldsymbol{v}}_h $$

and, for all $\underline{\boldsymbol{v}}_{w,h}\in \underline{\boldsymbol{W}}_{{{\,\mathrm{rot}\,}},h}^{k,k}$,

$$ \underline{{\mathcal{E}}}_{H^1,h}^k\partial _{{{\,\mathrm{rot}\,}},h}^k\underline{\boldsymbol{v}}_{w,h} \overset{(4.5)}{=} (\partial _{{{\,\mathrm{rot}\,}},h}^k\underline{\boldsymbol{v}}_{w,h},\underline{0}) \overset{(4.3)}{=} \underline{d}_{{{\,\mathrm{rot}\,}},h}^k(\underline{\boldsymbol{v}}_{w,h},\underline{0}) \overset{(4.5)}{=} \underline{d}_{{{\,\mathrm{rot}\,}},h}^k\underline{\boldsymbol{{\mathcal{E}}}}_{{{\,\mathrm{rot}\,}},h}^k\underline{\boldsymbol{v}}_{w,h}. $$

$$\square $$

### Interpolators

For all $T_2\in \mathcal{M}_{2,h}$, the interpolators on the spaces $\underline{V}_{{\textbf{grad}},T_2}^k$, $\underline{\boldsymbol{V}}_{{{\,\mathrm{rot}\,}},T_2}^k$ and $\underline{V}_{H^1,T_2}^k$ are such that, for smooth enough functions $q: T_2 \rightarrow \mathbb{R}$, $\boldsymbol{v}: T_2 \rightarrow \mathbb{R}^2$, and $r: T_2 \rightarrow \mathbb{R}$,

$$\begin{aligned} \underline{I}_{V,{\textbf{grad}},T_2}^{k} q&{:}{=}{{\underline{I}}}_{W,{\textbf{grad}},T_2}^{k-1,k}q, \\ \underline{\boldsymbol{I}}_{\boldsymbol{V}, {{\,\mathrm{rot}\,}},T_2}^k\boldsymbol{v}&{:}{=}\big ( \underline{\boldsymbol{I}}_{\boldsymbol{W},{{\,\mathrm{rot}\,}},T_2}^{k,k}, (\pi _{\mathcal{P},T_1}^{k-1}({{\,\mathrm{rot}\,}}\boldsymbol{v})_{T_1\in \mathcal{M}_{1,T_2}}, ({{\,\mathrm{rot}\,}}\boldsymbol{v}(\boldsymbol{x}_{T_0}))_{T_0\in \mathcal{M}_{0,T_2}} \big ), \\ \underline{I}_{V,H^1,T_2}^k r&{:}{=}\big (I_{W,L^2,T_2}^k , (\pi _{\mathcal{P},T_1}^{k-1} r)_{T_1\in \mathcal{M}_{1,T_2}}, (r(\boldsymbol{x}_{T_0}))_{T_0\in \mathcal{M}_{0,T_2}} \big ). \end{aligned} $$

By this definition, the interpolators $\underline{I}_{V,{\textbf{grad}},T_2}^{k}$, $\underline{\boldsymbol{I}}_{\boldsymbol{V}, {{\,\mathrm{rot}\,}},T_2}^k$ and $\underline{I}_{V,H^1,T_2}^k$ verify Assumption (C2).

### Serendipity rot-rot complex and homological properties

Lemma 12 and Theorem 14 ensure that the SDDR and rot-rot complexes satisfy Assumptions 1 and 2. We are now in a position to apply the construction (2.12) to the rot-rot complex in order to derive its serendipity version and characterize its cohomology.

#### Serendipity spaces and operators

Recalling (2.10), the serendipity version of spaces $\underline{V}_{{\textbf{grad}},h}^k$ and $\underline{\boldsymbol{V}}_{{{\,\mathrm{rot}\,}},h}^k$ can be written as follows:

4.11 $$\begin{aligned}&\widehat{\underline{V}}_{{\textbf{grad}},h}^k {:}{=}\underline{\widehat{W}}_{{\textbf{grad}},h}^{k} \\&\underline{\widehat{\boldsymbol{V}}}_{{{\,\mathrm{rot}\,}},h}^k {:}{=}\underline{\widehat{\boldsymbol{W}}}_{{{\,\mathrm{rot}\,}},h}^k\times {{\,\mathrm{Ker}\,}}\underline{\boldsymbol{{\mathcal{R}}}}_{{{\,\mathrm{rot}\,}},h}^k\cong \underline{\widehat{\boldsymbol{W}}}_{{{\,\mathrm{rot}\,}},h}^k\times \left( \times _{T_1\in \mathcal{M}_{1,h}}\mathcal{P}^{k-1}(T_1)\times \mathbb{R}^{\mathcal{M}_{0,h}} \right) . \end{aligned}$$

Accounting for the isomorphism in (4.11), we write a generic element $\underline{\widehat{\boldsymbol{v}}}_h$ of $\underline{\widehat{\boldsymbol{V}}}_{{{\,\mathrm{rot}\,}},h}^k$ as $\underline{\widehat{\boldsymbol{v}}}_h=\big (\underline{\widehat{\boldsymbol{v}}}_{w,h},\underline{v}_{\mathrm{c},h}\big )$ with $\underline{\widehat{\boldsymbol{v}}}_{w,h}\in \underline{\widehat{\boldsymbol{W}}}_{{{\,\mathrm{rot}\,}},h}^k$ and $\underline{v}_{\mathrm{c},h}$ such that $(\underline{\widehat{\boldsymbol{0}}},\underline{v}_{\mathrm{c},h})\in {{\,\mathrm{Ker}\,}}\underline{\boldsymbol{{\mathcal{R}}}}_{{{\,\mathrm{rot}\,}},h}^k$. We define the extension of $\underline{\widehat{\boldsymbol{W}}}_{{{\,\mathrm{rot}\,}},h}^k$ into $\underline{\widehat{\boldsymbol{V}}}_{{{\,\mathrm{rot}\,}},h}^k$ according to (2.12a):

$$ \underline{\widehat{\boldsymbol{{\mathcal{E}}}}}_{{{\,\mathrm{rot}\,}},h}^k\underline{\widehat{\boldsymbol{v}}}_{w,h} {:}{=}\big ( \underline{\widehat{\boldsymbol{v}}}_{w,h}, \underline{0} \big ). $$

The reduction is given by (2.12b):

$$ \underline{\widehat{\boldsymbol{{\mathcal{R}}}}}_{{{\,\mathrm{rot}\,}},h}^k(\underline{\widehat{\boldsymbol{v}}}_{w,h},\underline{v}_{\mathrm{c},h}) {:}{=}\underline{\widehat{\boldsymbol{v}}}_{w,h}. $$

The reduction operators $\underline{\widehat{R}}_{V,{\textbf{grad}},h}:\underline{V}_{{\textbf{grad}},h}^k\rightarrow \widehat{\underline{V}}_{{\textbf{grad}},h}^k$ and $\underline{\widehat{\boldsymbol{R}}}_{\boldsymbol{V},{{\,\mathrm{rot}\,}},h}:\underline{\boldsymbol{V}}_{{{\,\mathrm{rot}\,}},h}^k\rightarrow \underline{\widehat{\boldsymbol{V}}}_{{{\,\mathrm{rot}\,}},h}^k$ are defined using (2.12d) and accounting for the isomorphism (4.11): For all $\underline{q}_h\in \underline{V}_{{\textbf{grad}},h}^k$ and all $\underline{\boldsymbol{v}}_h\in \underline{\boldsymbol{V}}_{{{\,\mathrm{rot}\,}},h}^k$,

$$ \underline{\widehat{R}}_{V,{\textbf{grad}},h}\underline{q}_h {:}{=}\underline{\widehat{R}}_{\boldsymbol{W},{\textbf{grad}},h}\underline{q}_h \text{ and } \underline{\widehat{\boldsymbol{R}}}_{\boldsymbol{V},{{\,\mathrm{rot}\,}},h}\underline{\boldsymbol{v}}_h {:}{=}\Big (\underline{\widehat{\boldsymbol{R}}}_{\boldsymbol{W},{{\,\mathrm{rot}\,}},h}\underline{\boldsymbol{{\mathcal{R}}}}_{{{\,\mathrm{rot}\,}},h}^k\underline{\boldsymbol{v}}_h,\underline{v}_{\mathrm{c},h}\Big ), $$

with $\underline{\widehat{R}}_{\boldsymbol{W},{\textbf{grad}},h}$ and $\underline{\widehat{\boldsymbol{R}}}_{\boldsymbol{W},{{\,\mathrm{rot}\,}},h}$ respectively defined according to (3.13) and (3.14).

Finally, using (2.12c), the extension operators $\underline{E}_{V,{\textbf{grad}},h}:\widehat{\underline{V}}_{{\textbf{grad}},h}^k\rightarrow \underline{V}_{{\textbf{grad}},h}^k$ and $\underline{\boldsymbol{E}}_{\boldsymbol{V},{{\,\mathrm{rot}\,}},h}:\underline{\widehat{\boldsymbol{V}}}_{{{\,\mathrm{rot}\,}},h}^k\rightarrow \underline{\boldsymbol{V}}_{{{\,\mathrm{rot}\,}},h}^k$ are such that, for all $\underline{\widehat{q}}_h\in \widehat{\underline{V}}_{{\textbf{grad}},h}^k$ and all $(\underline{\widehat{\boldsymbol{v}}}_{w,h},\underline{v}_{\mathrm{c},h})\in \underline{\widehat{\boldsymbol{V}}}_{{{\,\mathrm{rot}\,}},h}^k$,

$$ \underline{E}_{V,{\textbf{grad}},h}\underline{\widehat{q}}_h {:}{=}\underline{E}_{W,{\textbf{grad}},h} \underline{\widehat{q}}_h \text{ and } \underline{\boldsymbol{E}}_{\boldsymbol{V},{{\,\mathrm{rot}\,}},h}\underline{\widehat{\boldsymbol{v}}}_h {:}{=}\underline{\boldsymbol{{\mathcal{E}}}}_{{{\,\mathrm{rot}\,}},h}^k\underline{\boldsymbol{E}}_{\boldsymbol{W},{{\,\mathrm{rot}\,}},h}\underline{\widehat{\boldsymbol{v}}}_{w,h} + (\underline{\widehat{\boldsymbol{0}}},\underline{v}_{\mathrm{c},h}), $$

with $\underline{E}_{W,{\textbf{grad}},h}$ and $\underline{\boldsymbol{E}}_{\boldsymbol{W},{{\,\mathrm{rot}\,}},h}$ respectively defined according to (3.11) and (3.12). For all $T_2\in \mathcal{M}_{2,h}$, the interpolators on the spaces $\widehat{\underline{V}}_{{\textbf{grad}},T_2}^k$ and $\underline{\widehat{\boldsymbol{V}}}_{{{\,\mathrm{rot}\,}},T_2}^k$ are such that, for smooth enough functions $q: T_2 \rightarrow \mathbb{R}$ and $\boldsymbol{v}: T_2 \rightarrow \mathbb{R}^2$,

$$\begin{aligned} \underline{{\widehat{I}}}_{V,{\textbf{grad}},T_2}^{k}q&{:}{=}{\underline{{\widehat{I}}}}_{W,{\textbf{grad}},T_2}^{k}q,\\ \underline{\widehat{\boldsymbol{I}}}_{\boldsymbol{V},{{\,\mathrm{rot}\,}},T_2}^k\boldsymbol{v}&{:}{=}\big (\underline{\widehat{\boldsymbol{I}}}_{\boldsymbol{W},{{\,\mathrm{rot}\,}},T_2}^{k}\boldsymbol{v}, (\pi _{\mathcal{P},T_1}^{k-1}({{\,\mathrm{rot}\,}}\boldsymbol{v})_{T_1\in \mathcal{M}_{1,T_2}}, ({{\,\mathrm{rot}\,}}\boldsymbol{v}(\boldsymbol{x}_{T_0}))_{T_0\in \mathcal{M}_{0,T_2}} \big ). \end{aligned} $$

According to this definition, we can write:

4.12 $$\begin{aligned} \underline{{\widehat{I}}}_{V,{\textbf{grad}},T_2}^{k}&=\underline{\widehat{R}}_{V,{\textbf{grad}},T_2}\underline{I}_{V,{\textbf{grad}},T_2}^{k}\end{aligned},$$

4.13 $$\begin{aligned} \underline{\widehat{\boldsymbol{I}}}_{\boldsymbol{V},{{\,\mathrm{rot}\,}},T_2}^k&=\underline{\widehat{\boldsymbol{R}}}_{\boldsymbol{V},{{\,\mathrm{rot}\,}},T_2}\underline{\boldsymbol{I}}_{\boldsymbol{V}, {{\,\mathrm{rot}\,}},T_2}^k \end{aligned}.$$

Using (2.11), the serendipity discrete differential operators are such that, for all $(\underline{\widehat{q}}_h,\underline{\widehat{\boldsymbol{v}}}_h)\in \widehat{\underline{V}}_{{\textbf{grad}},h}^k\times \underline{\widehat{\boldsymbol{V}}}_{{{\,\mathrm{rot}\,}},h}^k$:

$$ \begin{aligned}&\underline{\widehat{\boldsymbol{d}}}_{{\textbf{grad}},h}^k \underline{\widehat{q}}_h {:}{=}\big (\underline{\widehat{\boldsymbol{\partial }}}_{{\textbf{grad}},h}^{k}\underline{\widehat{q}}_h,\underline{0}\big ), \\&\underline{\widehat{d}}_{{{\,\mathrm{rot}\,}},h}^k\underline{\widehat{\boldsymbol{v}}}_h {:}{=}\big (\widehat{\partial }_{{{\,\mathrm{rot}\,}},h}^{k}\underline{\widehat{\boldsymbol{v}}}_{w,h},\underline{d}_{{{\,\mathrm{rot}\,}},h}^k(\underline{\widehat{\boldsymbol{0}}},\underline{v}_{\mathrm{c},h})\big )\overset{(4.3),\,(4.11)}{=}\big (\widehat{\partial }_{{{\,\mathrm{rot}\,}},h}^{k}\underline{\widehat{\boldsymbol{v}}}_{w,h},\underline{v}_{\mathrm{c},h}\big ). \end{aligned} $$

#### Serendipity rot-rot complex and isomorphism in cohomology

The serendipity rot-rot complex is given by:4.14

##### Theorem 15

(Homological properties of the complexes in (4.1)) All the complexes in the diagram (4.1) have cohomologies that are isomorphic to the cohomology of the continuous de Rham complex.

##### Proof

Lemma 12 and Theorem 14 ensure that Assumptions 1 and 2 are satisfied. We can therefore invoke Corollary 8 to infer that the cohomology of the Srot-rot complex (4.14) is isomorphic to the cohomology of the rot-rot complex (4.4), of the DDR2d complex (3.4), and, therefore, of the continuous de Rham complex. $\square$

##### Theorem 16

(Polynomial consistency of the serendipity rot-rot complex) The interpolators $\underline{{\widehat{I}}}_{V,{\textbf{grad}},T_2}^{k}$ and $\underline{\widehat{\boldsymbol{I}}}_{\boldsymbol{V},{{\,\mathrm{rot}\,}},T_2}^k$ verify the polynomial consistency property.

##### Proof

Lemma 13, Sect. 4.3, and equations (4.12), (4.13) ensure that Assumption 9 is satisfied so by Lemma 10, the interpolators on the rotrot and Srotrot spaces verify the polynomial consistency. $\square$

### Numerical examples

**Fig. 1:**
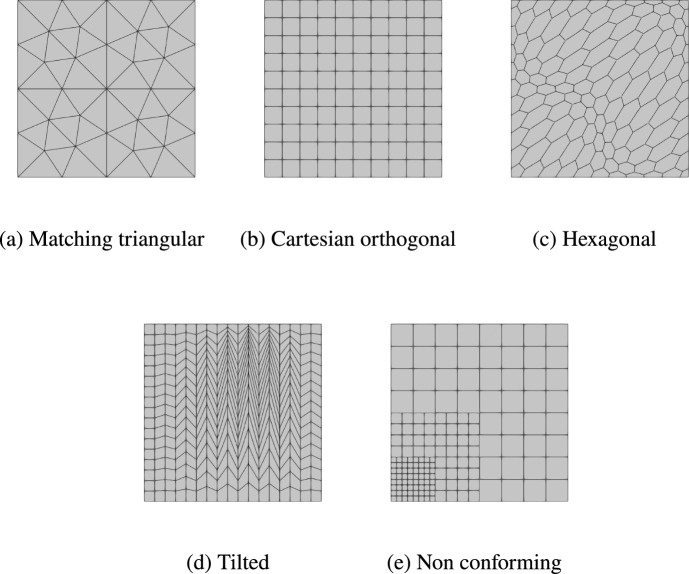
Meshes used in the numerical test

In order to show the effect of serendipity DOF reduction, we consider the quad-rot problem of [8, Section 5.2] and compare the results obtained using the original and serendipity spaces in terms of error versus dimension of the linear system (after elimination of Dirichlet DOFs) on different families of meshes. One mesh from each family is shown in Fig. 1.

In order to compare the serendipity and classical version of the scheme, we introduce the following measure of the error on the discrete spaces. Denoting respectively by $(\underline{\boldsymbol{u}},\underline{p})$ the exact solution and by $(\underline{\boldsymbol{u}}_h, \underline{p}_h)$ and $(\underline{\widehat{\boldsymbol{u}}}_h, \underline{\widehat{p}}_h)$ the numerical solutions obtained using standard and serendipity spaces, we define the following errors:

$$ \begin{aligned} \underline{\boldsymbol{e}}_{I,h}&{:}{=}\underline{\boldsymbol{u}}_h - \underline{\boldsymbol{I}}_{\boldsymbol{V}, {{\,\mathrm{rot}\,}},h}^k \boldsymbol{u}&\qquad \underline{\varepsilon }_{I,h}&{:}{=}\underline{p}_h - \underline{I}_{V,{\textbf{grad}},h}^{k} p, \\ \underline{\widehat{\boldsymbol{e}}}_{I,h}&{:}{=}\underline{\widehat{\boldsymbol{u}}}_h - \underline{\widehat{\boldsymbol{I}}}_{\boldsymbol{V},{{\,\mathrm{rot}\,}},h}^k \boldsymbol{u}&\qquad \underline{\widehat{\varepsilon }}_{I,h}&{:}{=}\underline{{\widehat{p}}}_h - \underline{{\widehat{I}}}_{V,{\textbf{grad}},h}^{k} p, \\ \underline{\boldsymbol{e}}_{{{\,\mathrm{rot}\,}},h}&{:}{=}\boldsymbol{R}_h^k\underline{R}_h^k\underline{\boldsymbol{u}}_h-{\textbf{rot}}{{\,\mathrm{rot}\,}}\boldsymbol{u}&\qquad \underline{\varepsilon }_{{\textbf{grad}},h}&{:}{=}\boldsymbol{G}_h^k \underline{p}_h - \nabla p, \\ \underline{\widehat{\boldsymbol{e}}}_{{{\,\mathrm{rot}\,}},h}&{:}{=}\widehat{\boldsymbol{R}}_h^k\underline{{\widehat{R}}}_h^k\underline{\widehat{\boldsymbol{u}}}_h-{\textbf{rot}}{{\,\mathrm{rot}\,}}\boldsymbol{u}&\qquad \underline{\widehat{\varepsilon }}_{{\textbf{grad}},h}&{:}{=}\widehat{\boldsymbol{G}}_h^k \underline{{\widehat{p}}}_h - \nabla p. \end{aligned} $$

where $\underline{I}_{V,{\textbf{grad}},h}^{k}$, $\underline{{\widehat{I}}}_{V,{\textbf{grad}},h}^{k}$, $\underline{\boldsymbol{I}}_{\boldsymbol{V}, {{\,\mathrm{rot}\,}},h}^k$, and $\underline{\widehat{\boldsymbol{I}}}_{\boldsymbol{V},{{\,\mathrm{rot}\,}},h}^k$ respectively denote the interpolators on $\underline{V}_{{\textbf{grad}},h}^k$, $\widehat{\underline{V}}_{{\textbf{grad}},h}^k$, $\underline{\boldsymbol{V}}_{{{\,\mathrm{rot}\,}},h}^k$, and $\underline{\widehat{\boldsymbol{V}}}_{{{\,\mathrm{rot}\,}},h}^k$, and where the operators $\boldsymbol{G}_h^k$, $\widehat{\boldsymbol{G}}_h^k$, $\boldsymbol{R}_h^k\underline{R}_h^k$, and $\widehat{\boldsymbol{R}}_h^k\underline{{\widehat{R}}}_h^k$ are respectively the discrete gradient and the discrete rot-rot from the spaces $\underline{V}_{{\textbf{grad}},h}^k$, $\widehat{\underline{V}}_{{\textbf{grad}},h}^k$, $\underline{\boldsymbol{V}}_{{{\,\mathrm{rot}\,}},h}^k$, and $\underline{\widehat{\boldsymbol{V}}}_{{{\,\mathrm{rot}\,}},h}^k$.

The errors are measured by $$ L^2$$ and $$L^2$$-like operator norms defined in the spirit of [11, Section 4.4] and, consistently with [8], respectively denoted by $$\Vert \cdot \Vert _{V,h}$$ for $$\underline{V}_{{\textbf{grad}},h}^k$$ and $$\widehat{\underline{V}}_{{\textbf{grad}},h}^k$$ and $$\Vert \cdot \Vert _{\boldsymbol{\Sigma },h}$$ for $$\underline{\boldsymbol{V}}_{{{\,\mathrm{rot}\,}},h}^k$$ and $$\underline{\widehat{\boldsymbol{V}}}_{{{\,\mathrm{rot}\,}},h}^k$$ (we do not distinguish the notation for the norms on the standard and serendipity spaces, as they have formally the same expression and the exact meaning is made clear by the argument). On the latter spaces, we additionally consider the norm $$\Vert \cdot \Vert _{{\textbf{rot}}{{\,\mathrm{rot}\,}},h}$$, an $$L^2$$-like norm of the discrete rot-rot operator defined as in [8, Eq. (4.29)]. The problem data, meshes, and polynomial degrees are almost the same as in the above reference, with two new mesh sequences [Fig. 1d, e], so we do not repeat these details here, while the number of edges $$\eta _{T_1}$$ for each edge $$T_1 \in \mathcal{M}_{h,1}$$ is chosen the same way as in [12]. The aforementioned errors use norms on the discrete spaces. Although the norms are essentially the same, the serendipity and non-serendipity scheme have different mass matrices. To ensure that the preservation of the accuracy of the serendipity scheme is not a mere consequence of using a discrete norm, we introduce the following errors computed with respect to the continuous solution:

$$ \begin{aligned} \underline{\boldsymbol{e}}_{P,h}&{:}{=}\underline{\boldsymbol{P}}_{V,{{\,\mathrm{rot}\,}},h}^k \underline{\boldsymbol{u}}_h-\boldsymbol{u}&\qquad \underline{\varepsilon }_{P,h}&{:}{=}\underline{P}_{V,{\textbf{grad}},h}^{k+1} \underline{p}_h -p, \\ \underline{\widehat{\boldsymbol{e}}}_{P,h}&{:}{=}\underline{\widehat{\boldsymbol{P}}}_{V,{{\,\mathrm{rot}\,}},h}^k \underline{\widehat{\boldsymbol{u}}}_h-\boldsymbol{u}&\qquad \underline{\widehat{\varepsilon }}_{P,h}&{:}{=}\underline{{\widehat{P}}}_{V,{\textbf{grad}},h}^{k+1} \underline{{\widehat{p}}}_h -p, \end{aligned} $$

where $\underline{\boldsymbol{P}}_{V,{{\,\mathrm{rot}\,}},h}^k$ and $\underline{P}_{V,{\textbf{grad}},h}^{k+1}$ are potential reconstruction operators defined such that: For all $\underline{\boldsymbol{v}}_{T_2}\in \underline{\boldsymbol{V}}_{{{\,\mathrm{rot}\,}},T_2}^k$,

$$\begin{aligned} \int _{T_2}\underline{\boldsymbol{P}}_{V,{{\,\mathrm{rot}\,}},T_2}^k\underline{\boldsymbol{v}}_{T_2}\cdot ({\textbf{rot}}q + \boldsymbol{w}) = \int _TR_T^k\underline{\boldsymbol{v}}_T~q + \sum _{T_1\in \mathcal{M}_{1,{T_2}}}\omega _{T_2T_1}\int _{T_1} v_{T_1}~q + \int _{T_2}\boldsymbol{v}_{\boldsymbol{\mathcal{R}},T}^\mathrm{c}\cdot \boldsymbol{w} \\ \forall (q,\boldsymbol{w})\in \mathcal{P}^{k+1,0}(T)\times \boldsymbol{\mathcal{R}}^{\mathrm{c},k}(T) \end{aligned}$$

and, for all $\underline{q}_{T_2}\in \underline{V}_{{\textbf{grad}},T_2}^k$,

$$ \int _{T_2} \underline{P}_{V,{\textbf{grad}},T_2}^{k+1} \underline{q}_{T_2}~{{\,\mathrm{div}\,}}\boldsymbol{v} = -\int _{T_2}\boldsymbol{G}_{T_2}^k\underline{q}_{T_2}\cdot \boldsymbol{v} + \sum _{T_1\in \mathcal{M}_{1,{T_2}}}\omega _{T_2T_1}\int _{T_1} {P_{V,T_1}^{k+1}}\underline{q}_{T_1}~(\boldsymbol{v}\cdot \boldsymbol{n}_{T_1}) \qquad \forall \boldsymbol{v}\in \boldsymbol{\mathcal{R}}^{\mathrm{c},k+2}(T). $$

with $${P_{V,T_1}^{k+1}} $$ is the unique polynomial that satisfies $$P_{V,T_1}^{k+1}\underline{q}_{T_1}(\boldsymbol{x}_\nu ) = q_\nu $$ for all $$\nu \in \mathcal{M}_{0,T_1}$$ and $$\pi _{\mathcal{P},T_1}^{k-1}(P_{V,E}^{k+1}\underline{q}_{T_1}) = q_{T_1}$$.

**Fig. 2:**
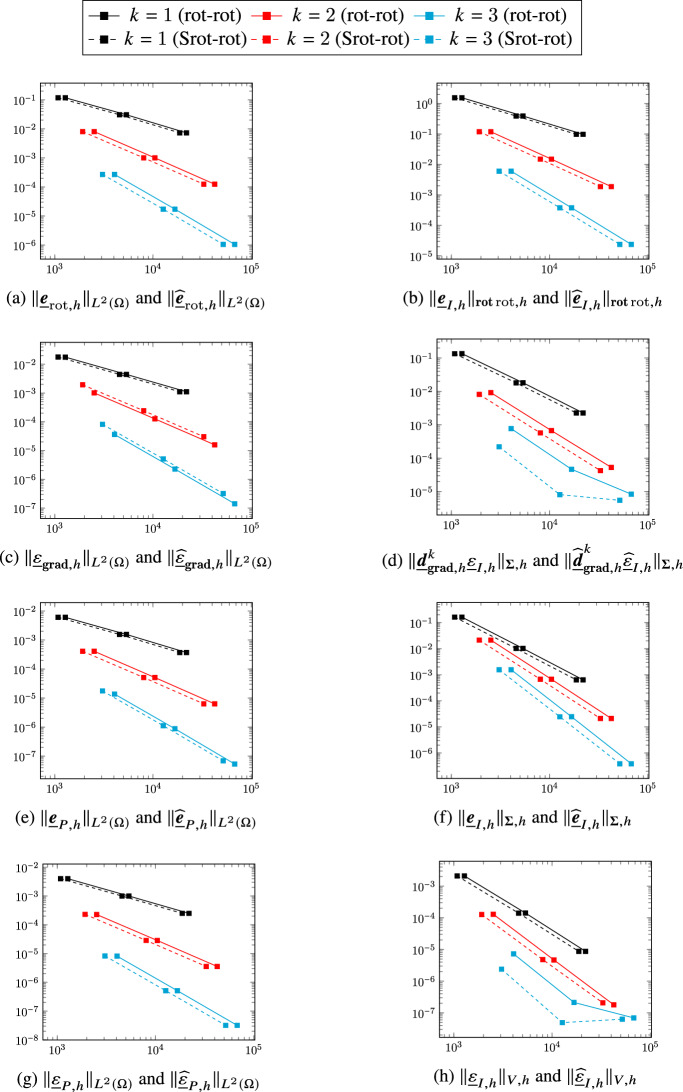
Errors norm vs. linear system size using the standard (continuous lines) and serendipity spaces (dashed lines) to solve the quad-rot problem of [8, Section 5.2] on the Cartesian orthogonal mesh family

**Fig. 3:**
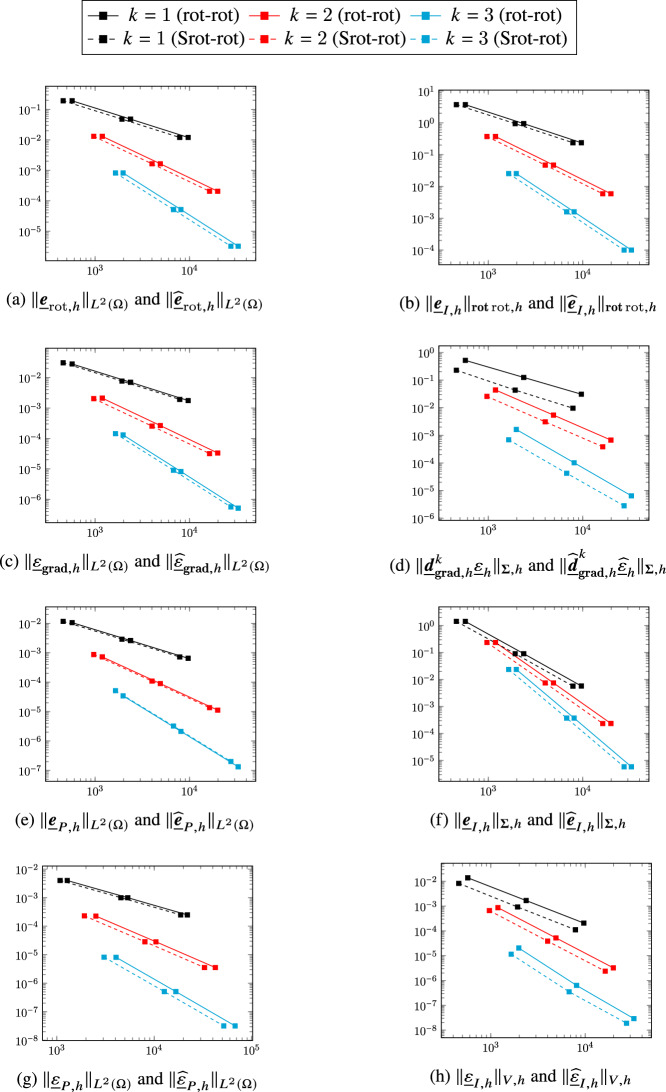
Errors norm vs. linear system sizeusing the standard (continuous lines) and serendipity spaces (dashed lines) to solve the quad-rot problem of [8, Section 5.2] on the triangular mesh family.

**Fig. 4:**
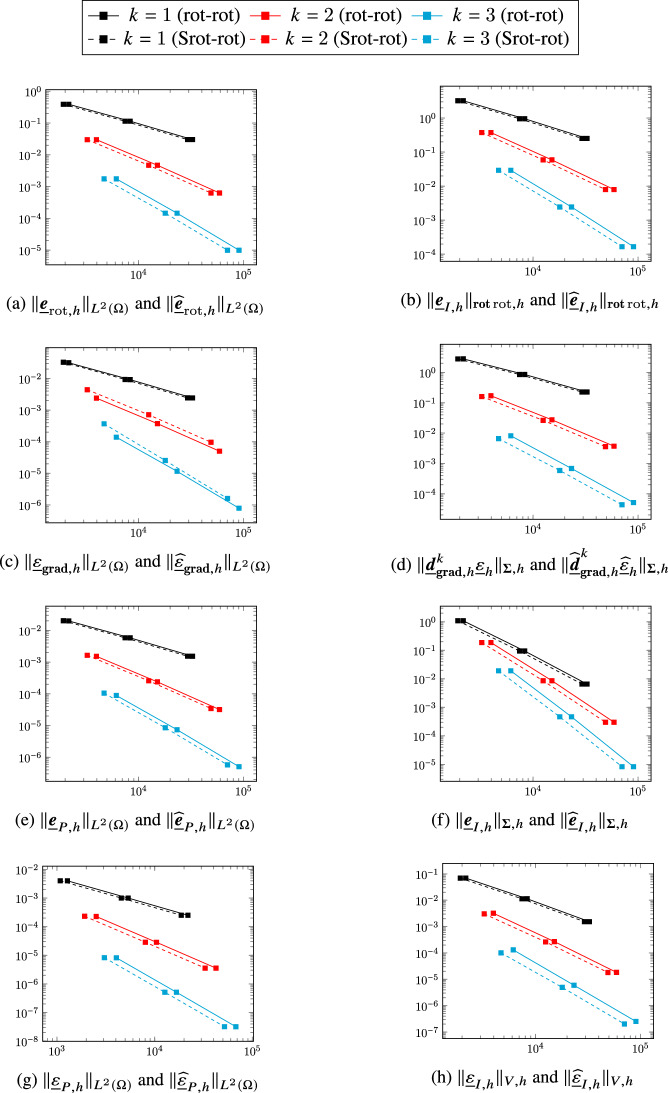
Errors norm vs. linear system size using the standard (continuous lines) and serendipity spaces (dashed lines) to solve the quad-rot problem of [8, Section 5.2] on the hexagonal mesh family.

**Fig. 5:**
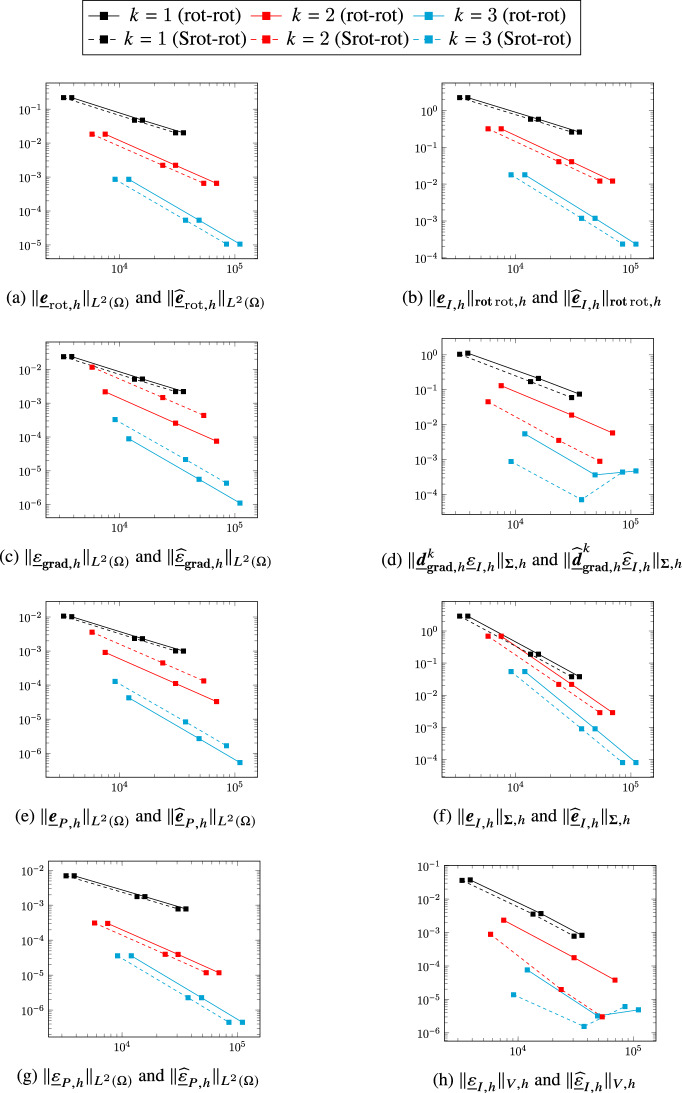
Errors norm vs. linear system size using the standard (continuous lines) and serendipity spaces (dashed lines) to solve the quad-rot problem of [8, Section 5.2] on the Tilted Squares mesh family.

**Fig. 6:**
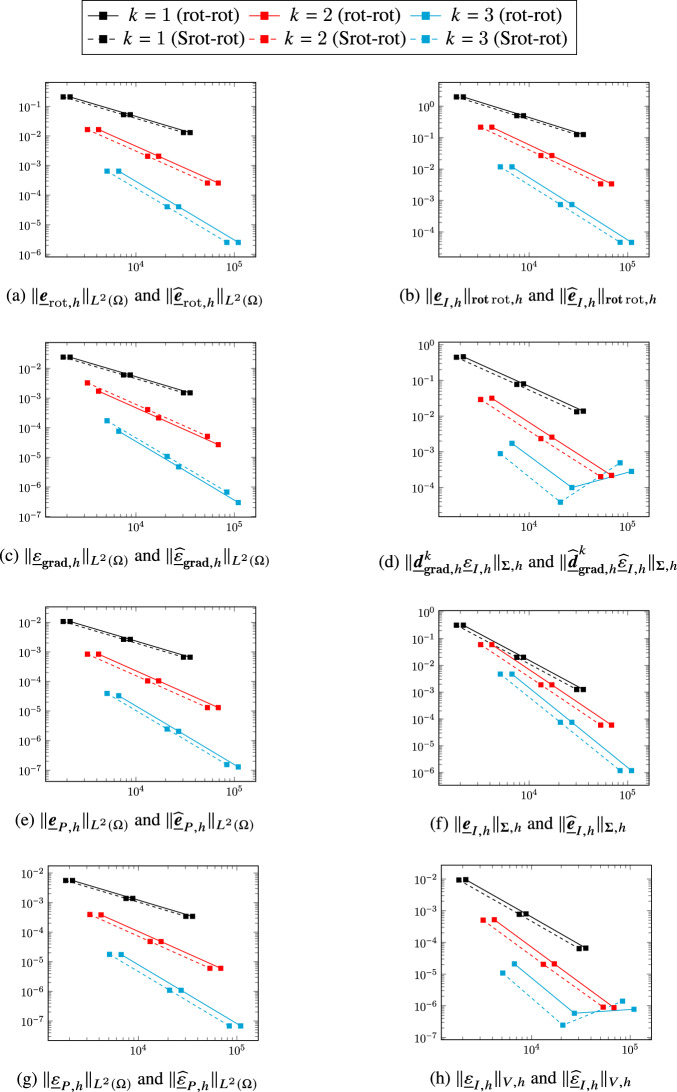
Errors norm vs. linear system size using the standard (continuous lines) and serendipity spaces (dashed lines) to solve the quad-rot problem of [8, Section 5.2] on the Non conforming squares mesh family

The various error measures displayed in Figs. 2, 3, 4, 5 and 6 show that a given precision is invariably obtained with fewer DOFs using serendipity spaces, the more so the higher the degree. In some cases, we observe a smaller error with the serendipity spaces than with the non-serendipity for the same mesh and degree. This phenomenon is most notably noticeable for the errors ${\Vert \underline{\widehat{\boldsymbol{d}}}_{{\textbf{grad}},h}^k\underline{\widehat{\varepsilon }}_{I,h}\Vert _{\boldsymbol{\Sigma },h}}$, and $\Vert \underline{\widehat{\varepsilon }}_{I,h}\Vert _{V,h}$ in Figs. 2, 5 and 6. This is explained by the use of discrete norms that are equivalent as the mesh size tends to zero but not strictly equal. However, this feature does not affect the general behavior of the solutions. This is further confirmed comparing the serendipity solution and the non-serendipity one on the same continuous space.

A comparison in terms of error versus meshsize *h*, not reported here for the sake of conciseness, shows that the serendipity and non-serendipity schemes yield essentially the same error for a given mesh and polynomial degree, with visible differences only for the pressure errors $\Vert \underline{\varepsilon }_{I,h}\Vert _{V,h}$, $\Vert \underline{\widehat{\varepsilon }}_{I,h}\Vert _{V,h}$, $\Vert \underline{\varepsilon }_{{\textbf{grad}},h}\Vert _{\widehat{\boldsymbol{\Sigma }},h}$, and $\Vert \underline{\widehat{\varepsilon }}_{{\textbf{grad}},h}\Vert _{\widehat{\boldsymbol{\Sigma }},h}$ for $k=3$. We also notice that a stagnation of the errors ${\Vert \underline{\varepsilon }_{{\textbf{grad}},h}\Vert _{\boldsymbol{\Sigma },h}}$/${\Vert \underline{\widehat{\varepsilon }}_{{\textbf{grad}},h}\Vert _{\boldsymbol{\Sigma },h}}$ $\Vert \underline{\varepsilon }_h\Vert _{V,h}$/$\Vert \underline{\widehat{\varepsilon }}_h\Vert _{V,h}$ can be observed for the finest meshes in Figs. 2, 5 and 6. This behaviour is most likely related to the fast degradation of the condition number for fourth-order problems, and could likely be improved using appropriate preconditioners.

## A serendipity Stokes complex

In this section we discuss a second application of the general construction considering the three-dimensional Stokes complex (1.3). Diagram (2.6) specialized to the present case becomes5.1The top horizontal portion of this diagram corresponds to (3.16). In the rest of this section we will provide precise definitions of the remaining spaces and operators involved in the construction.

### Discrete Stokes complex

We will start by giving a brief overview of the construction of a discrete counterpart of the complex (1.3) developed in [19].

#### Discrete spaces

For each edge $T_1 \in \mathcal{M}_{1,h}$, we will need the following space spanned by vector-valued polynomial functions that are normal to $T_1$:

$$ \boldsymbol{\mathcal{P}}_{\boldsymbol{n}}^{k}(T_1) {:}{=}\left\{ p_1 \boldsymbol{n}_1 + p_2 \boldsymbol{n}_2 \,:\,p_1, p_2 \in \mathcal{P}^{k}(T_1) \right\} , $$

where $\boldsymbol{n}_1$ and $\boldsymbol{n}_2$ are two arbitrary orthogonal unit vectors normal to $T_1$. The discrete counterparts of the spaces $H^2(\Omega )$, $\boldsymbol{H}^1({\textbf{curl}};\Omega )$, $\boldsymbol{H}^1(\Omega )$, and $L^2(\Omega )$ read:

$$ \underline{V}_{{\textbf{grad}},h}^k {:}{=}\underline{W}_{{\textbf{grad}},h}^{k-1,k-1,k} \times \underline{V}_{{\textbf{grad}},\mathrm{c},h}^k,\quad \underline{\boldsymbol{V}}_{{\textbf{curl}},h}^k {:}{=}\underline{\boldsymbol{W}}_{{\textbf{curl}},h}^{k,k,k} \times \underline{\boldsymbol{V}}_{{\textbf{curl}},\mathrm{c},h}^k,\quad \underline{\boldsymbol{V}}_{{{\,\mathrm{div}\,}},h}^k {:}{=}\underline{\boldsymbol{W}}_{{{\,\mathrm{div}\,}},h}^{k} \times \underline{\boldsymbol{V}}_{{{\,\mathrm{div}\,}},\mathrm{c},h}^k $$

where the additional components with respect to the standard three-dimensional DDR spaces are given by

$$\begin{aligned} \underline{V}_{{\textbf{grad}},\mathrm{c},h}^k&{:}{=}\times _{T_2\in \mathcal{M}_{2,h}}\mathcal{P}^{k-1}(T_2) \times \times _{T_1\in \mathcal{M}_{1,h}}\boldsymbol{\mathcal{P}}_{\boldsymbol{n}}^{k}(T_1)\times \mathbb{R}^{3\mathcal{M}_{0,h}}, \\ \underline{\boldsymbol{V}}_{{\textbf{curl}},\mathrm{c},h}^k&{:}{=}\begin{aligned}&\times _{T_2\in \mathcal{M}_{2,h}}\left( \mathcal{P}^{k-1}(T_2)\times \boldsymbol{\mathcal{G}}^{k}(T_2)\times \boldsymbol{\mathcal{G}}^{\mathrm{c},k}(T_2) \right) \\&\times \times _{T_1\in \mathcal{M}_{1,h}}\left( \boldsymbol{\mathcal{P}}^{k+1}(T_1;\mathbb{R}^3)\times \boldsymbol{\mathcal{P}}_{\boldsymbol{n}}^{k}(T_1) \right) \times \left( \mathbb{R}^{3\mathcal{M}_{0,h}} \right) ^2, \end{aligned} \\ \underline{\boldsymbol{V}}_{{{\,\mathrm{div}\,}},\mathrm{c},h}^k&{:}{=}\times _{T_2\in \mathcal{M}_{2,h}}\left( \boldsymbol{\mathcal{G}}^{k}(T_2)\times \boldsymbol{\mathcal{G}}^{\mathrm{c},k}(T_2) \right) \times \times _{T_1\in \mathcal{M}_{1,h}}\widetilde{\mathcal{P}}^{k+3}(T_1;\mathbb{R}^3), \end{aligned}$$

where, to write $\underline{\boldsymbol{V}}_{{\textbf{curl}},h}^k$, we have decomposed the space $\widetilde{\mathcal{P}}^{k+2}(\mathcal{M}_{1,h};\mathbb{R}^3)$ in [19, Definition (3.3)] as $\times _{T_1\in \mathcal{M}_{1,h}}\left( \mathcal{P}^{k}(T_1)\times \boldsymbol{\mathcal{P}}_{\boldsymbol{n}}^{k}(T_1)\right) \times \mathbb{R}^{3\mathcal{M}_{0,h}}$ and $\widetilde{\mathcal{P}}^{m}(T_1;\mathbb{R}^3)$ denotes the space of vector-valued functions over $T_1$ whose components are in $\mathcal{P}^{m}(T_1)$ and are continuous on $T_1$.

#### Discrete gradient

Let $$\underline{q}_h=(\underline{q}_{w,h},\underline{q}_{\mathrm{c},h})\in \underline{V}_{{\textbf{grad}},h}^k$$ with

$$ \underline{q}_{\mathrm{c},h} {:}{=}\left( (G_{q,T_2})_{T_2\in \mathcal{M}_{2,h}}, (\boldsymbol{G}_{q,T_1})_{T_1\in \mathcal{M}_{1,h}}, (\boldsymbol{G}_{q,T_0})_{T_0\in \mathcal{M}_{0,h}} \right) \in \underline{V}_{{\textbf{grad}},\mathrm{c},h}^k, $$

where $G_{q,T_2}$, $\boldsymbol{G}_{q,T_1}$, and $\boldsymbol{G}_{q,T_0}$ have, respectively, the meaning of a normal gradient to the face $T_2$, a normal gradient to the edge $T_1$, and a full gradient at the vertex $T_0$. The DDR discrete gradient is completed to map from $\underline{V}_{{\textbf{grad}},h}^k$ to $\underline{\boldsymbol{V}}_{{\textbf{curl}},h}^k$ by adding the following component:

$$ \begin{aligned} \underline{\boldsymbol{d}}_{{\textbf{grad}},\mathrm{c},h}^k\underline{q}_{\mathrm{c},h}{:}{=}\Big (&(G_{q,T_2}, \boldsymbol{\pi }_{\boldsymbol{\mathcal{G}},T_2}^{k} \boldsymbol{RG}^k_{T_2} \underline{q}_{\mathrm{c},T_2}, \boldsymbol{\pi }_{\boldsymbol{\mathcal{G}},T_2}^{\mathrm{c},k} \boldsymbol{RG}^k_{T_2} \underline{q}_{\mathrm{c},T_2})_{T_2\in \mathcal{M}_{2,h}}, \\&(\boldsymbol{G}_{q,T_1},\boldsymbol{v}_{T_1}'\times \boldsymbol{t}_{T_1})_{T_1\in \mathcal{M}_{1,h}}, \\&(\boldsymbol{G}_{q,T_0},\boldsymbol{0})_{T_0\in \mathcal{M}_{0,h}} \Big ) \in \underline{\boldsymbol{V}}_{{\textbf{curl}},\mathrm{c},h}^k, \end{aligned} $$

where $\underline{q}_{\mathrm{c},T_2}$ is the restriction of $\underline{q}_{\mathrm{c},h}$ to the elements neighbooring $T_2$, $\boldsymbol{RG}^k_{T_2}$ is the rotor of the normal gradient defined by

$$ \int _{T_2} \boldsymbol{RG}^k_{T_2} \underline{q}_{T_2} \cdot \boldsymbol{w} = - \int _{T_2} G_{q,T_2} {{\,\mathrm{rot}\,}}\boldsymbol{w} - \sum _{T_1 \in \mathcal{M}_{1,T_2}} \omega _{T_2T_1} \int _{T_1} (\boldsymbol{G}_{q,T_1} \cdot \boldsymbol{n}_{T_2} ) (\boldsymbol{w} \cdot \boldsymbol{t}_{T_1}) \quad \forall \boldsymbol{w} \in \boldsymbol{\mathcal{P}}^{k}(T_2), $$

and $\boldsymbol{v}_{T_1}'$ is the derivative along the edge $T_1$ of the function $\boldsymbol{v}_{T_1}$ such that $\boldsymbol{\pi }^k_{\boldsymbol{\mathcal{P}},T_1}\boldsymbol{v}_{T_1} = \boldsymbol{G}_{q,{T_1}}$ and for all $T_0\in \mathcal{M}_{0,T_1}$, $\boldsymbol{v}_{T_1}(\boldsymbol{x}_{T_0}) = \boldsymbol{G}_{q,T_0}$. The discrete gradient $\underline{\boldsymbol{d}}_{{\textbf{grad}},{h}}^k: \underline{V}_{{\textbf{grad}},h}^k \rightarrow \underline{\boldsymbol{V}}_{{\textbf{curl}},h}^k$ is then given by

5.2 $$\begin{aligned} \underline{\boldsymbol{d}}_{{\textbf{grad}},{h}}^k~\underline{q}_h{:}{=}\left( \underline{\boldsymbol{\partial }}_{{\textbf{grad}},h}^k\underline{q}_{w,h},\underline{\boldsymbol{d}}_{{\textbf{grad}},\mathrm{c},h}^k\underline{q}_{\mathrm{c},h} \right) . \end{aligned}$$

#### Discrete curl

For $\underline{\boldsymbol{v}}_h=(\underline{\boldsymbol{v}}_{w,h},\underline{\boldsymbol{v}}_{\mathrm{c},h})\in \underline{\boldsymbol{V}}_{{\textbf{curl}},h}^k$, the component $\underline{\boldsymbol{v}}_{\mathrm{c},h}$ is given by

$$ \underline{\boldsymbol{v}}_{\mathrm{c},h}{:}{=}\left( (v_{T_2},\boldsymbol{R}_{v,\boldsymbol{\mathcal{G}},{T_2}},\boldsymbol{R}^c_{v,\boldsymbol{\mathcal{G}},T_2})_{T_2\in \mathcal{M}_{2,h}}, (\boldsymbol{R}_{v,T_1},\boldsymbol{v}_{n,T_1})_{T_1\in \mathcal{M}_{1,h}}, (\boldsymbol{v}_{T_0}, \boldsymbol{R}_{v,T_0})_{T_0\in \mathcal{M}_{0,h}} \right) \in \underline{\boldsymbol{V}}_{{\textbf{curl}},\mathrm{c},h}^k, $$

where $v_{T_2}$, $(\boldsymbol{R}_{v,\boldsymbol{\mathcal{G}},{T_2}},\boldsymbol{R}^c_{v,\boldsymbol{\mathcal{G}},T_2})$, $\boldsymbol{R}_{v,T_1}$, and $(v_{T_0}\boldsymbol{R}_{v,T_0})$ have, respectively, the meaning of the normal flux accross the face $T_2$, the normal gradient of the tangential components to the face $T_2$, the tangential component of the curl plus the normal gradient of the tangential component to the edge $T_1$, and the value of the function and of its curl at the vertex $T_0$. The discrete curl in the DDR complex (3.8) is completed by adding the following component in order to obtain a map from $\underline{\boldsymbol{V}}_{{\textbf{curl}},h}^k$ to $\underline{\boldsymbol{V}}_{{{\,\mathrm{div}\,}},h}^k$:

$$ \begin{aligned} \underline{\boldsymbol{d}}_{{\textbf{curl}},\mathrm{c},h}^k\underline{\boldsymbol{v}}_{\mathrm{c},h}{:}{=}\Big (&(\boldsymbol{\pi }_{\boldsymbol{\mathcal{G}},T_2}^{k} \boldsymbol{C}^k_{T_2} \underline{\boldsymbol{v}}_{\mathrm{c},T_2},\boldsymbol{R}_{v,\boldsymbol{\mathcal{G}},T_2}, \boldsymbol{\pi }_{\boldsymbol{\mathcal{G}},T_2}^{\mathrm{c},k}\boldsymbol{C}^k_{T_2} \underline{\boldsymbol{v}}_{\mathrm{c},T_2},\boldsymbol{R}^c_{v,\boldsymbol{\mathcal{G}},T_2})_{T_2\in \mathcal{M}_{2,h}}, \\&(\boldsymbol{C}^k_{T_1} \underline{\boldsymbol{v}}_{\mathrm{c},T_1})_{T_1\in \mathcal{M}_{1,h}} \Big ) \in \underline{\boldsymbol{V}}_{{{\,\mathrm{div}\,}},\mathrm{c},h}^k, \end{aligned} $$

where $\underline{\boldsymbol{v}}_{\mathrm{c},T_2}$ is the restriction of $\underline{\boldsymbol{v}}_{\mathrm{c},h}$ to the elements sharing $T_2$, $\underline{\boldsymbol{v}}_{\mathrm{c},T_1}$ the restriction of $\underline{\boldsymbol{v}}_{\mathrm{c},h}$ to the elements sharing $T_1$, $\boldsymbol{C}^k_{T_2}$ is the face curl defined in (3.3), and $\boldsymbol{C}^k_{T_1}$ is such that $\boldsymbol{C}^k_{T_1} \underline{\boldsymbol{v}}_{\mathrm{c},T_1} (\boldsymbol{x}_{T_0})= \boldsymbol{R}_{v,T_0}$ and $\boldsymbol{\pi }_{\boldsymbol{\mathcal{P}},T_1}^{k+1}\boldsymbol{C}^k_{T_1} \underline{\boldsymbol{v}}_{\mathrm{c},T_1}= \boldsymbol{R}_{v,T_1}- \boldsymbol{v}_{\boldsymbol{n},T_1}' ~\times ~{\boldsymbol{t}}_{T_1}$, with $\boldsymbol{v}_{\boldsymbol{n},T_1}$ such that $\boldsymbol{\pi }_{\boldsymbol{\mathcal{P}},T_1}^{k}\boldsymbol{v}_{\boldsymbol{n},T_1} = \boldsymbol{v}_{n,T_1}$ and for all $T_0\in \mathcal{M}_{0,T_1}$, $\boldsymbol{v}_{\boldsymbol{n},T_1}(\boldsymbol{x}_{T_0}) = \boldsymbol{v}_{T_0}$. The discrete curl is then given by

5.3 $$\begin{aligned} \underline{\boldsymbol{d}}_{{\textbf{curl}},{h}}^k~\underline{\boldsymbol{v}}_h&{:}{=}\left( \underline{\boldsymbol{\partial }}_{{\textbf{curl}},h}^{k}\underline{\boldsymbol{v}}_{w,h},\underline{\boldsymbol{d}}_{{\textbf{curl}},\mathrm{c},h}^k\underline{\boldsymbol{v}}_{\mathrm{c},h} \right) . \end{aligned}$$

#### Discrete divergence

The discrete divergence is nothing but the original DDR divergence defined by (3.9) but with domain $\underline{\boldsymbol{V}}_{{{\,\mathrm{div}\,}},h}^k$ instead of $\underline{\boldsymbol{W}}_{{{\,\mathrm{div}\,}},h}^{k}$: For all $\underline{\boldsymbol{w}}_h=(\underline{\boldsymbol{w}}_{w,h},\underline{\boldsymbol{w}}_{\mathrm{c},h})\in \underline{\boldsymbol{V}}_{{{\,\mathrm{div}\,}},h}^k$,

$$ d_{{{\,\mathrm{div}\,}},h}^k~\underline{\boldsymbol{w}}_h {:}{=}\partial _{{{\,\mathrm{div}\,}},h}^k\underline{\boldsymbol{w}}_{w,h}. $$

#### Discrete Stokes complex

The discrete counterpart of the Stokes complex (1.3) which appears at the bottom and back of diagram (5.1) is given by:5.4

### Extension and reduction maps between the three-dimensional DDR and Stokes complexes

We next define extension and reduction operators between the three-dimensional DDR complex (3.8) and the discrete Stokes complex (5.4) that satisfy Assumption 2. The proof is similar to that of Theorem 14 and is omitted for the sake of brevity. It follows once again from Remark 3 that (3.8) and (5.4) have isomorphic cohomologies.

The extension operators are such that: For all $\underline{q}_{w,h}\in \underline{W}_{{\textbf{grad}},h}^{k-1,k-1,k}$, all $\underline{\boldsymbol{v}}_{w,h}\in \underline{\boldsymbol{W}}_{{\textbf{curl}},h}^{k,k,k}$, and all $\underline{\boldsymbol{w}}_{w,h}\in \underline{\boldsymbol{W}}_{{{\,\mathrm{div}\,}},h}^{k}$,

$$ \underline{{\mathcal{E}}}_{{\textbf{grad}},h}^k\underline{q}_{w,h} {:}{=}\big ( \underline{q}_{w,h},\underline{0}\big ),\quad \underline{\boldsymbol{{\mathcal{E}}}}_{{\textbf{curl}},h}^k\underline{\boldsymbol{v}}_{w,h} {:}{=}\big ( \underline{\boldsymbol{v}}_{w,h},\underline{\boldsymbol{0}}\big ),\quad \underline{\boldsymbol{{\mathcal{E}}}}_{{{\,\mathrm{div}\,}},h}^k\underline{\boldsymbol{w}}_{w,h} {:}{=}\big ( \underline{\boldsymbol{w}}_{w,h},\underline{\boldsymbol{0}}\big ). $$

The reduction map is such that, for all $\underline{q}_h=(\underline{q}_{w,h},\underline{q}_{\mathrm{c},h})\in \underline{V}_{{\textbf{grad}},h}^k$, all $\underline{\boldsymbol{v}}_h=(\underline{\boldsymbol{v}}_{w,h},\underline{\boldsymbol{v}}_{\mathrm{c},h})\in \underline{\boldsymbol{V}}_{{\textbf{curl}},h}^k$, and all $\underline{\boldsymbol{w}}_h=(\underline{\boldsymbol{w}}_{w,h},\underline{\boldsymbol{w}}_{\mathrm{c},h})\in \underline{\boldsymbol{V}}_{{{\,\mathrm{div}\,}},h}^k$,

$$ \underline{{\mathcal{R}}}_{{\textbf{grad}},h}^k\underline{q}_h {:}{=}\underline{q}_{w,h},\quad \underline{\boldsymbol{{\mathcal{R}}}}_{{\textbf{curl}},h}^k\underline{\boldsymbol{v}}_h {:}{=}\underline{\boldsymbol{v}}_{w,h},\quad \underline{\boldsymbol{{\mathcal{R}}}}_{{{\,\mathrm{div}\,}},h}^k\underline{\boldsymbol{w}}_h {:}{=}\underline{\boldsymbol{w}}_{w,h}. $$

For future reference, we note the following isomorphisms, which are a direct consequence of the above definitions:

5.5 $$\begin{aligned} \, {{\,\mathrm{Ker}\,}}\underline{{\mathcal{R}}}_{{\textbf{grad}},h}^k\cong \underline{V}_{{\textbf{grad}},\mathrm{c},h}^k \mathrm{and} {{\,\mathrm{Ker}\,}}\underline{\boldsymbol{{\mathcal{R}}}}_{{\textbf{curl}},h}^k\cong \underline{\boldsymbol{V}}_{{\textbf{curl}},\mathrm{c},h}^k. \end{aligned}$$

### Interpolators

For all $T_3\in \mathcal{M}_{3,h}$, the interpolators on the spaces $\underline{V}_{{\textbf{grad}},T_3}^k$, $\underline{\boldsymbol{V}}_{{\textbf{curl}},T_3}^k$, and $\underline{\boldsymbol{V}}_{{{\,\mathrm{div}\,}},T_3}^k$ are such that, for smooth enough functions $q: T_3 \rightarrow \mathbb{R}$, $\boldsymbol{v}: T_3 \rightarrow \mathbb{R}^3$, and $\boldsymbol{w}: T_3 \rightarrow \mathbb{R}^3$,

$$ \begin{aligned} \underline{I}_{V,{\textbf{grad}},T_3}^{k} q&{:}{=}\big ({{\underline{I}}}_{W,{\textbf{grad}},T_3}^{k-1,k-1,k}, \Pi _{{\textbf{grad}},c,T_3}q \big ), \\ \underline{\boldsymbol{I}}_{V,{\textbf{curl}},T_3}^{k}\boldsymbol{v}&{:}{=}\big ( \underline{\boldsymbol{I}}_{\boldsymbol{W},{\textbf{curl}},T_3}^{k,k,k},\boldsymbol{\Pi }_{{\textbf{curl}},c,T_3}\boldsymbol{v}\big ), \\ \underline{\boldsymbol{I}}_{V,{{\,\mathrm{div}\,}},T_3}^{k} \boldsymbol{w}&{:}{=}\big (\underline{\boldsymbol{I}}_{\boldsymbol{W},{{\,\mathrm{div}\,}},T_3}^{k},\boldsymbol{\Pi }_{{{\,\mathrm{div}\,}},c,T_3}\boldsymbol{w}\big ), \end{aligned} $$

where

$$ \begin{aligned} \Pi _{{\textbf{grad}},c,T_3}(q)&{:}{=}\big ( (\pi _{\mathcal{P},T_2}^{k-1}({\textbf{grad}}(q)\cdot \boldsymbol{n}_{T_2}))_{T_2\in \mathcal{M}_{2,T_3}}, (\boldsymbol{\pi }_{\boldsymbol{\mathcal{P}},T_1}^{k-1}(\boldsymbol{t}_{T_1} \times ({\textbf{grad}}(q)\times \boldsymbol{t}_{T_1})))_{T_1\in \mathcal{M}_{1,T_3}},\\&\quad ({\textbf{grad}}q(\boldsymbol{x}_{T_0}))_{T_0\in \mathcal{M}_{0,T_3}}\big ), \\ \Pi _{{\textbf{curl}},c,T_3}(\boldsymbol{v})&{:}{=}\big ( (\boldsymbol{\pi }_{\boldsymbol{\mathcal{G}},T_2}^{k}(\boldsymbol{n}_{T_2}\times (\nabla \boldsymbol{v} \cdot \boldsymbol{n}_{T_2})), \boldsymbol{\pi }_{\boldsymbol{\mathcal{G}},T_2}^{\mathrm{c},k}(\boldsymbol{n}_{T_2}\times (\nabla \boldsymbol{v} \cdot \boldsymbol{n}_{T_2})), \pi _{\mathcal{P},T_2}^{k-1}(\boldsymbol{v}\cdot \boldsymbol{n}_{T_2}) )_{T_2\in \mathcal{M}_{2,T_3}},\\&\quad (\boldsymbol{\pi }_{\boldsymbol{\mathcal{P}},T_1}^{k+1}(({\textbf{curl}}\boldsymbol{v} \cdot \boldsymbol{t}_{T_1})\boldsymbol{t}_{T_1} + {\textbf{grad}}(\boldsymbol{v}\cdot \boldsymbol{t}_{T_1}) \times \boldsymbol{t}_{T_1} ))_{T_1\in \mathcal{M}_{1,T_3}}, ({\textbf{curl}}\boldsymbol{v}(\boldsymbol{x}_{T_0}))_{T_0\in \mathcal{M}_{0,T_3}}\big ), \\ \Pi _{{{\,\mathrm{div}\,}},c,T_3}(\boldsymbol{w})&{:}{=}\big ( (\boldsymbol{\pi }_{\boldsymbol{\mathcal{G}},T_2}^{k}(\boldsymbol{w}_{t,T_2}), \boldsymbol{\pi }_{\boldsymbol{\mathcal{G}},T_2}^{\mathrm{c},k}(\boldsymbol{w}_{t,T_2}) )_{T_2\in \mathcal{M}_{2,T_3}}, (\boldsymbol{\pi }_{\boldsymbol{\mathcal{P}},T_1}^{k+1}(\boldsymbol{w}))_{T_1\in \mathcal{M}_{1,T_3}}, (\boldsymbol{w}(\boldsymbol{x}_{T_0}))_{T_0\in \mathcal{M}_{0,T_3}}\big ). \end{aligned} $$

The components of the interpolators mapping into the kernel of the reduction maps are precisely $\Pi _{\bullet ,c,T_3}$ for $\bullet \in \left\{ {\textbf{grad}},{\textbf{curl}},{{\,\mathrm{div}\,}}\right\}$. This explicit decomposition readily allows to infer that these interpolators satisfy Assumption (C2).

### Serendipity Stokes complex and homological properties

Applying the construction of Sect. 2 to the Stokes complex and recalling the isomorphisms (5.5), we obtain the following serendipity version of the spaces $\underline{V}_{{\textbf{grad}},h}^k$ and $\underline{\boldsymbol{V}}_{{\textbf{curl}},h}^k$:

5.6 $$\begin{aligned} \widehat{\underline{V}}_{{\textbf{grad}},h}^k {:}{=}\underline{\widehat{W}}_{{\textbf{grad}},h}^{k} \times \underline{V}_{{\textbf{grad}},\mathrm{c},h}^k,\quad \underline{\widehat{\boldsymbol{V}}}_{{\textbf{curl}},h}^k {:}{=}\underline{\widehat{\boldsymbol{W}}}_{{\textbf{curl}},h}^k \times \underline{\boldsymbol{V}}_{{\textbf{curl}},\mathrm{c},h}^k, \end{aligned}$$

where $\underline{\widehat{W}}_{{\textbf{grad}},h}^{k}$ and $\underline{\widehat{\boldsymbol{W}}}_{{\textbf{curl}},h}^k$ are the serendipity DDR spaces defined by (3.10).

We write generic elements $\widehat{\underline{q}}_h$ of $\widehat{\underline{V}}_{{\textbf{grad}},h}^k$ and $\underline{\widehat{\boldsymbol{v}}}_h$ of $\underline{\widehat{\boldsymbol{V}}}_{{\textbf{curl}},h}^k$ respectively as $\widehat{\underline{q}}_h=(\widehat{\underline{q}}_{w,h},{\underline{q}}_{\mathrm{c},h})$ and $\underline{\widehat{\boldsymbol{v}}}_h=(\underline{\widehat{\boldsymbol{v}}}_{w,h},\underline{\boldsymbol{v}}_{\mathrm{c},h})$ with $\widehat{\underline{q}}_{w,h}\in \underline{\widehat{W}}_{{\textbf{grad}},h}^{k}$, $\underline{\widehat{\boldsymbol{v}}}_{w,h}\in \underline{\widehat{\boldsymbol{W}}}_{{\textbf{curl}},h}^k$, and ${\underline{q}}_{\mathrm{c},h} \in \underline{V}_{{\textbf{grad}},\mathrm{c},h}^k$ and $\underline{\boldsymbol{v}}_{\mathrm{c},h} \in \underline{\boldsymbol{V}}_{{\textbf{curl}},\mathrm{c},h}^k$.

According to (2.12a), we define the extensions of the SDDR spaces into serendipity Stokes spaces as follows: For all $\widehat{\underline{q}}_{w,h} \in \underline{\widehat{W}}_{{\textbf{grad}},h}^{k}$ and all $\underline{\widehat{\boldsymbol{v}}}_{w,h} \in \underline{\widehat{\boldsymbol{W}}}_{{\textbf{curl}},h}^k$,

$$ \widehat{\underline{{\mathcal{E}}}}_{{\textbf{grad}},h}^k\widehat{\underline{q}}_{w,h} {:}{=}\big ( \widehat{\underline{q}}_{w,h}, {\underline{0}} \big ) \text{ and } \underline{\widehat{\boldsymbol{{\mathcal{E}}}}}_{{\textbf{curl}},h}^k\underline{\widehat{\boldsymbol{v}}}_{w,h} {:}{=}\big ( \underline{\widehat{\boldsymbol{v}}}_{w,h}, \underline{\boldsymbol{0}} \big ). $$

The reduction map between the SStokes and the SDDR complexes is given by (2.12b): For all $(\widehat{\underline{q}}_{w,h}, {\underline{q}}_{\mathrm{c},h}) \in \widehat{\underline{V}}_{{\textbf{grad}},h}^k$ and all $(\underline{\widehat{\boldsymbol{v}}}_{w,h},\underline{\boldsymbol{v}}_{\mathrm{c},h}) \in \underline{\widehat{\boldsymbol{V}}}_{{\textbf{curl}},h}^k$,

$$ \widehat{\underline{{\mathcal{R}}}}_{{\textbf{grad}},h}^k(\widehat{\underline{q}}_{w,h},{\underline{q}}_{\mathrm{c},h}) {:}{=}\widehat{\underline{q}}_{w,h} \text{ and } \underline{\widehat{\boldsymbol{{\mathcal{R}}}}}_{{\textbf{curl}},h}^k(\underline{\widehat{\boldsymbol{v}}}_{w,h},\underline{\boldsymbol{v}}_{\mathrm{c},h}) {:}{=}\underline{\widehat{\boldsymbol{v}}}_{w,h}. $$

By (2.12d), the reduction map from the Stokes to the SStokes complexes are given by: For all $\underline{q}_h = (\underline{q}_{w,h}, \underline{q}_{\mathrm{c},h})\in \underline{V}_{{\textbf{grad}},h}^k$ and all $\underline{\boldsymbol{v}}_h = (\underline{\boldsymbol{v}}_{w,h}, \underline{\boldsymbol{v}}_{\mathrm{c},h}) \in \underline{\boldsymbol{V}}_{{{\,\mathrm{rot}\,}},h}^k$,

$$ \widehat{\underline{R}}_{V,{\textbf{grad}},h}\underline{q}_h {:}{=}\Big (\underline{\widehat{R}}_{\boldsymbol{W},{\textbf{grad}},h}\underline{{\mathcal{R}}}_{{\textbf{grad}},h}^k\underline{q}_h, \underline{q}_{\mathrm{c},h} \Big ) \text{ and } \underline{\widehat{\boldsymbol{R}}}_{V,{\textbf{curl}},h}\underline{\boldsymbol{v}}_h {:}{=}\Big ( \underline{\widehat{\boldsymbol{R}}}_{\boldsymbol{W},{\textbf{curl}},h}\underline{\boldsymbol{{\mathcal{R}}}}_{{\textbf{curl}},h}^k\underline{\boldsymbol{v}}_h, \underline{\boldsymbol{v}}_{\mathrm{c},h} \Big ). $$

The extension operators from the SStokes to the Stokes complexes are defined according to (2.12c): For all $\widehat{\underline{q}}_h\in \widehat{\underline{V}}_{{\textbf{grad}},h}^k$ and all $\underline{\widehat{\boldsymbol{v}}}_h\in \underline{\widehat{\boldsymbol{V}}}_{{\textbf{curl}},h}^k$,

$$ \begin{aligned} \underline{E}_{V,{\textbf{grad}},h}\widehat{\underline{q}}_h&{:}{=}\underline{{\mathcal{E}}}_{{\textbf{grad}},h}^k\underline{E}_{W,{\textbf{grad}},h} \widehat{\underline{q}}_{w,h}+(\widehat{\underline{0}},{\underline{q}}_{\mathrm{c},h}), \\ \underline{\boldsymbol{E}}_{V,{\textbf{curl}},h}\underline{\widehat{\boldsymbol{v}}}_h&{:}{=}\underline{\boldsymbol{{\mathcal{E}}}}_{{\textbf{curl}},h}^k\underline{\boldsymbol{E}}_{\boldsymbol{W},{\textbf{curl}},h}\underline{\widehat{\boldsymbol{v}}}_{w,h} + (\underline{\widehat{\boldsymbol{0}}},\underline{\boldsymbol{v}}_{\mathrm{c},h}). \end{aligned} $$

For all $T_3\in \mathcal{M}_{3,h}$, the interpolators on the spaces $\widehat{\underline{V}}_{{\textbf{grad}},T_3}^k$ and $\underline{\widehat{\boldsymbol{V}}}_{{\textbf{curl}},T_3}^k$ are such that, for smooth enough functions $q: T_3 \rightarrow \mathbb{R}$ and $\boldsymbol{v}: T_3 \rightarrow \mathbb{R}^3$,

$$\begin{aligned} \underline{{\widehat{I}}}_{V,{\textbf{grad}},T_3}^{k}q&{:}{=}\big ({\underline{{\widehat{I}}}}_{W,{\textbf{grad}},T_3}^{k}q,\Pi _{{\textbf{grad}},c,T_3}q\big ),\\ \underline{\widehat{\boldsymbol{I}}}_{V,{\textbf{curl}},T_3}^{k}\boldsymbol{v}&{:}{=}\big (\underline{\widehat{\boldsymbol{I}}}_{\boldsymbol{W},{\textbf{curl}},T_3}^{k}\boldsymbol{v},\boldsymbol{\Pi }_{{\textbf{curl}},c,T_3}\boldsymbol{v}\big ). \end{aligned} $$

According to this definition, we can write:

5.7 $$\begin{aligned} \underline{{\widehat{I}}}_{V,{\textbf{grad}},T_3}^{k}&=\widehat{\underline{R}}_{V,{\textbf{grad}},h}\underline{I}_{V,{\textbf{grad}},T_3}^{k} \end{aligned},$$

5.8 $$\begin{aligned} \underline{\widehat{\boldsymbol{I}}}_{V,{\textbf{curl}},T_3}^{k}&=\underline{\widehat{\boldsymbol{R}}}_{V,{\textbf{curl}},h}\underline{\boldsymbol{I}}_{V,{\textbf{curl}},T_3}^{k} \end{aligned}.$$

Using (2.11), the serendipity discrete differential operators are such that, for all $(\widehat{\underline{q}}_h,\underline{\widehat{\boldsymbol{v}}}_h)\in \widehat{\underline{V}}_{{\textbf{grad}},h}^k\times \underline{\widehat{\boldsymbol{V}}}_{{\textbf{curl}},h}^k$,

$$\begin{aligned} \underline{\widehat{\boldsymbol{d}}}_{{\textbf{grad}},h}^k \widehat{\underline{q}}_h&{:}{=}\big (\underline{\widehat{\boldsymbol{\partial }}}_{{\textbf{grad}},h}^{k}\widehat{\underline{q}}_h,\underline{\boldsymbol{d}}_{{\textbf{grad}},{h}}^k (\widehat{\underline{0}},{\underline{q}}_{\mathrm{c},h})\big )\overset{\mathrm{(5.2)},\mathrm{(5.6)}}{=}\big (\underline{\widehat{\boldsymbol{\partial }}}_{{\textbf{grad}},h}^{k}\widehat{\underline{q}}_h,\underline{\boldsymbol{d}}_{{\textbf{grad}},\mathrm{c},h}^k {\underline{q}}_{\mathrm{c},h}\big ),\\ \underline{\widehat{\boldsymbol{d}}}_{{\textbf{curl}},h}^k\underline{\widehat{\boldsymbol{v}}}_h&{:}{=}\big (\underline{\widehat{\boldsymbol{\partial }}}_{{\textbf{curl}},h}^{k}\underline{\widehat{\boldsymbol{v}}}_{w,h},\underline{\boldsymbol{d}}_{{\textbf{curl}},{h}}^k(\underline{\widehat{\boldsymbol{0}}},\underline{\boldsymbol{v}}_{\mathrm{c},h})\big )\overset{\mathrm{(5.3)},\mathrm{(5.6)}}{=}\big (\underline{\widehat{\boldsymbol{\partial }}}_{{\textbf{curl}},h}^{k}\underline{\widehat{\boldsymbol{v}}}_{w,h},\underline{\boldsymbol{d}}_{{\textbf{curl}},\mathrm{c},h}^k \underline{\boldsymbol{v}}_{\mathrm{c},h}\big ). \end{aligned}$$

This completes the definition of the serendipity Stokes complex corresponding to the bottom front complex in diagram (5.1). The following theorem can be proved using arguments similar to Theorem 15. The details are omitted for the sake of brevity.

#### Theorem 17

(Homological properties of the complexes in (5.1)) All the complexes in the diagram (5.1) have cohomologies that are isomorphic to the cohomology of the continuous de Rham complex.

#### Theorem 18

(Polynomial consistency of the serendipity Stokes complex) The interpolators $\underline{{\widehat{I}}}_{V,{\textbf{grad}},T_3}^{k}$ and $\underline{\widehat{\boldsymbol{I}}}_{V,{\textbf{curl}},T_3}^{k}$ verify the polynomial consistency property.

#### Proof

Lemma 13, Sect. 5.3 and Eqs. (5.7), (5.8) ensure that Assumption 9 is satisfied so by Lemma 10, the interpolators on the Stokes and serendipity Stokes spaces verify the polynomial consistency. $\square$
